# Interdependence of coagulation with immunotherapy and BRAF/MEK inhibitor therapy: results from a prospective study

**DOI:** 10.1007/s00262-024-03850-y

**Published:** 2024-11-02

**Authors:** Malte Beckmann, Julian Schlüter, Michael Erdmann, Rafaela Kramer, Sarah Cunningham, Holger Hackstein, Robert Zimmermann, Lucie Heinzerling

**Affiliations:** 1 Department of Dermatology, Comprehensive Cancer Center Erlangen-European Metropolitan Area of Nuremberg (CCC ER-EMN), University Hospital Erlangen, Deutsches Zentrum Immuntherapie (DZI), Friedrich-Alexander-University Erlangen-Nuremberg (FAU), 91054 Erlangen, Germany; 2 Department of Transfusion Medicine and Hemostaseology, University Hospital Erlangen, Friedrich-Alexander University Erlangen-Nuremberg (FAU), 91054 Erlangen, Germany; 3 Department of Dermatology and Allergology, LMU University Hospital Munich, Ludwig-Maximilian University, Frauenlobstraße 9 – 11, 80337 Munich, Germany

**Keywords:** Immune checkpoint inhibitors, BRAF/MEK inhibitors, Melanoma, Coagulation–fibrinolysis disorders, ICI-associated thrombosis, Predictive marker

## Abstract

Immune checkpoint inhibitor (ICI) therapies effectively treat a broadening spectrum of cancer entities but induce various immune-related side effects (irAEs). Recent reports suggest a correlation between ICI-induced systemic inflammation and thromboembolic events as well as an increased effectiveness by coadministration of anticoagulants. With cancer patients having a higher risk of thrombotic events per se, it is crucial to dissect and characterize the mechanisms that cause pro-coagulative effects induced by systemic tumor therapies and their potential interplay with anti-tumor response. A total of 31 patients with advanced skin cancer treated with either ICIs (*n* = 24) or BRAF/MEK inhibitors (*n* = 7) were longitudinally assessed for blood and coagulation parameters before as well as 7, 20 and 40 days after initiation of systemic tumor therapy. Changes were analyzed and compared between both groups. In addition, the influence of coagulation parameters on progression-free, recurrence-free and overall survival was investigated. The ICI cohort presented significantly increased factor VIII activity after one week of therapy (*p* 0.0225); while, protein S activity was reduced during the whole observation period. Additionally, von Willebrand factor activity and tissue factor concentrations increased under immunotherapy. Similar changes occurred under BRAF/MEK inhibitor therapy (BRAF/MEKi). Increased baseline levels of von Willebrand factor antigen and factor VIII:C before the start of ICI therapy correlated with a significantly higher risk of recurrence for patients receiving adjuvant immunotherapy. The findings suggest the induction of a pro-coagulant state under ICI and BRAF/MEKi and a role of coagulation parameters in the efficacy of ICI therapies.

**Supplementary Information:**

The online version contains supplementary material available at 10.1007/s00262-024-03850-y.

## Introduction

Immune checkpoint inhibitor (ICI) and targeted BRAF/MEK inhibitor therapy (BRAF/MEKi) have dramatically improved the outcome of patients with metastatic melanoma [1] with ICIs being effective in many other cancer entities including non-small-cell lung cancer (NSCLC), urothelial cancer, squamous cell carcinoma and Merkel cell carcinoma. By blocking inhibitory pathways, ICI-containing therapy regimes induce a variety of immune-related adverse events (irAEs) mainly affecting skin, endocrine glands, digestive tract, liver, lungs or joints, but can potentially occur in any tissue [2]. Furthermore, hematological immune-related adverse events (hem-irAEs) such as thrombocytopenia, leukocytopenia, anemia and rarely hemophagocytic lymphohistiocytosis have been described [3, 4]. Coagulopathies including an acquired factor V inhibition and the induction of hemophilia have been described in single cases [5, 6]. Additionally, severe thromboembolic or hemorrhagic events were reported during anti-PD-1/PD-L1 antibody treatment, suggesting a correlation between a systemic immune activation and an elevated incidence of coagulation–fibrinolysis disorders during the therapy with ICIs [7–10]. Those were preferentially described shortly after treatment initiation of systemic tumor therapy and in patients with high PD-L1-expression [11]. A recent case control study showed an increased atherosclerotic plaque formation under ICI therapy [12].

The most common side effects with combined BRAF/MEKi are pyrexia, nausea, rash, chills, diarrhea and hypertension [13]. Although less frequent, an association between BRAF/MEKi and cardiovascular or hematological side effects such as heart failure (reported odds ratio [ROR] = 1.62 compared to BRAF inhibitor monotherapy), venous (ROR = 1.8) and pulmonary thromboembolism (ROR = 1.91) or anemia (occurred in 10.5–22% of the patients treated) have been demonstrated [13, 14].

Importantly, cancer patients have a significantly higher risk of both arterial (ATE, hazard ratio [HR]: 2.2), venous thromboembolism (VTE, [HR]: 4–9), and pulmonary embolism [15–17]. This is due to the increased production and release of pro-coagulant substances such as tissue factor (TF), which promote activation of factor Xa and thus stimulate and enhance the coagulation cascade. This, combined with the additional release of pro-inflammatory cytokines and the fibrinolysis-inhibiting plasminogen activator inhibitor-1 (PAI-1) from tumor cells, promotes the formation of pathological thrombi on vascular endothelial cells [18]. Pro-inflammatory states have been described for ICI patients, but also for patients treated with BRAF/MEKi [19, 20]. As a clinical manifestation of these, inflammatory side effects including fever, diarrhea, pneumonitis, colitis and immune-induced endocrine dysfunctions occur regularly [2, 13]. Increased production and release of pro-inflammatory cytokines by immune cells is suspected as a potential mechanism of this pro-inflammatory status and associated irAEs under ICI therapy. For example, increased levels of IL-6, IL-10, IL-1β and IL-17 have already been associated with different irAEs [21]. Furthermore, a reduced incidence of irAEs under ICI therapy combined with the IL-6 receptor antibody tocilizumab has already been shown in a clinical study [22].

With a 4–9 times higher risk compared to the general population [16, 17], VTEs are the second most prevalent source of death in cancer patients [23]. Recent studies suggest a correlation between the systemic inflammatory effect of ICI therapy and an increase in thromboembolic events (TEE) [12, 24, 25]. VTEs occurred in 1.5–2.7% and ATEs in 1.1% of ICI-treated patients [24].

So far, it is controversial whether the incidence of thromboembolism differs from that in patients treated with conventional chemotherapy [26]. Patients, who developed VTEs under ICI therapy had a significantly worse prognosis with a median survival of 365 days compared to 453 in patients without VTE (hazard ratio = 1.22) [27].

Thus, studies to characterize the mechanisms and potentially even quantify the individual risk for thromboembolic events in patients undergoing immunotherapy or targeted therapy are needed. This prospective two-armed clinical study investigates changes in a wide range of coagulation and blood parameters upon initiation of ICI vs. BRAF/MEKi. The study aimed to detect whether and how these systemic tumor therapies induce a tendency to a pro-coagulant state and what influence this state has on the outcome.

## Patients and methods

### Study design and patient involvement

**Fig. 1:**
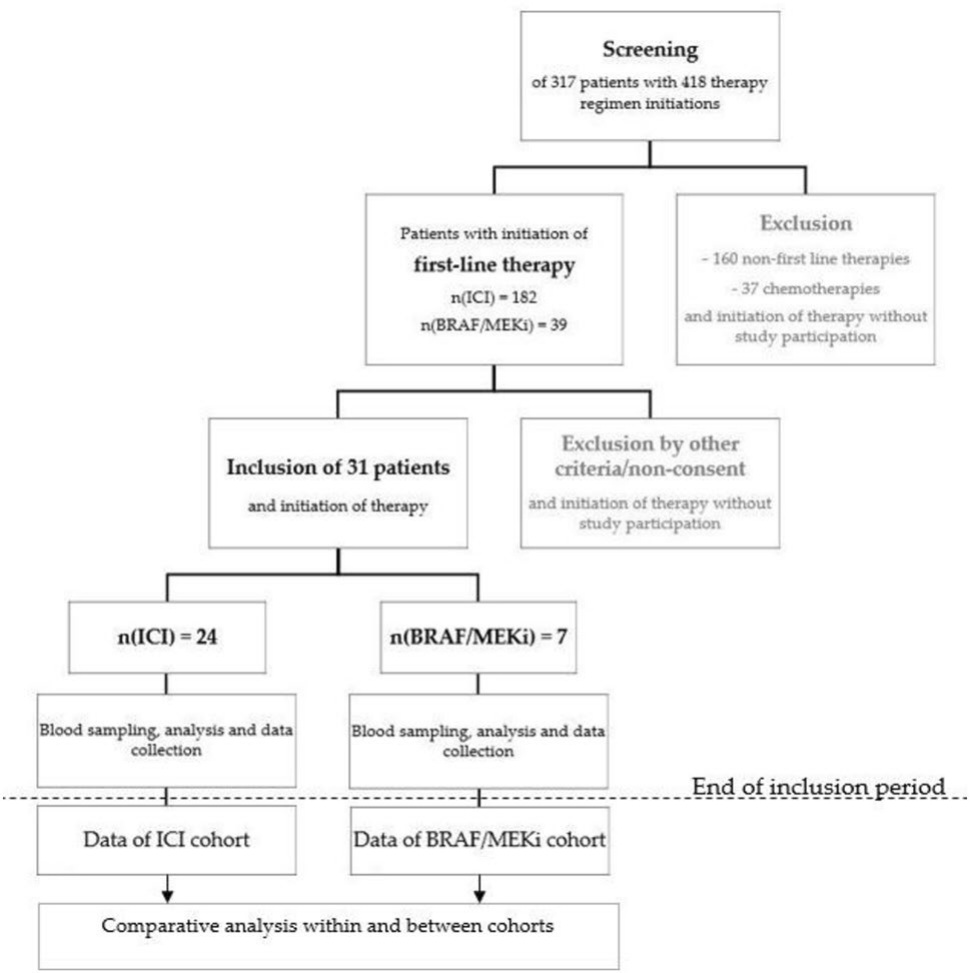
Study design During the study inclusion period from April 2019 to March 2021, a total of 418 treatment regimens were initiated in 317 patients. Patients were excluded from the study if they were not planned for either ICI or BRAF/MEKi therapy, if they already received one of the two therapies, were regularly taking coagulation-altering medication or had known coagulation–fibrinolysis disorders. The planned therapy was administered and data collected according to the study protocol. After data collection was complete, data within and between cohorts was analyzed with a comparative statistical analysis of the mean values via mixed-effects analysis with Geisser-Greenhouse correction and Tukey’s test for multiple comparisons

**Fig. 2:**
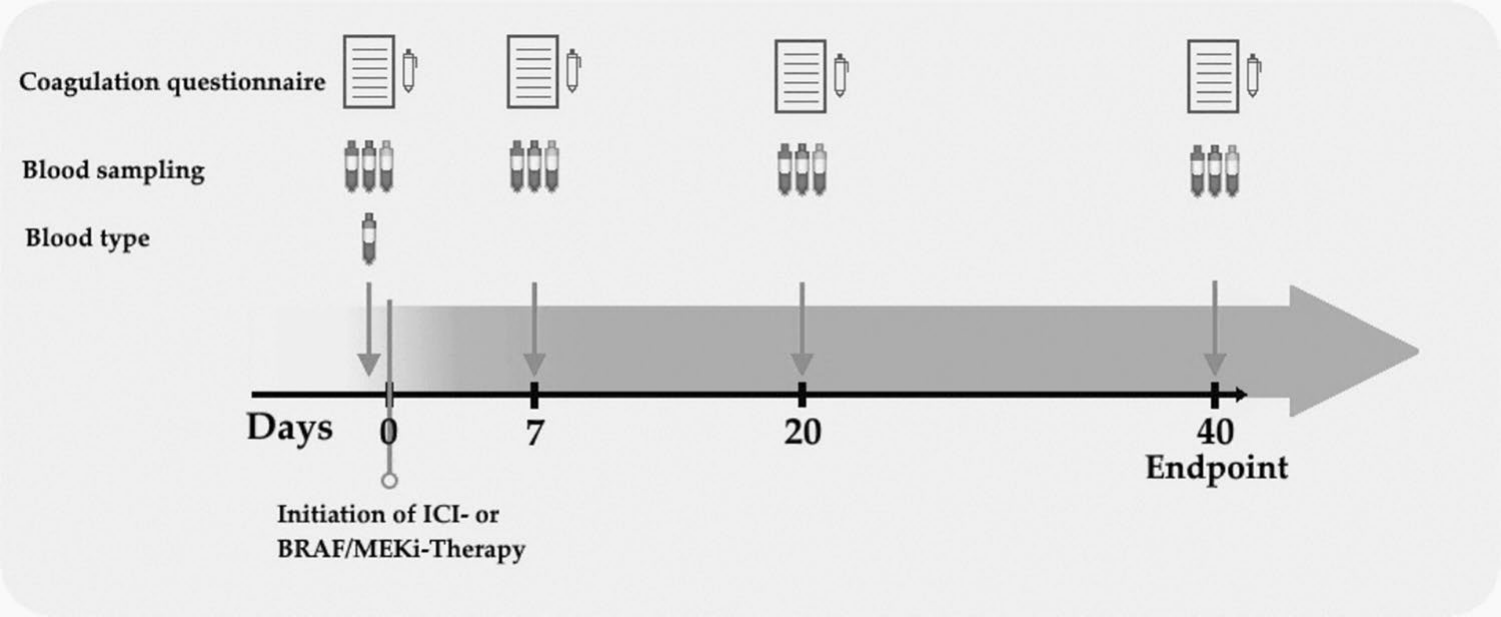
Study procedure Patients were included after giving informed consent. Blood samples were taken at baseline (t0) and 7, 20 and 40 days after therapy initiation (t7, t20 and t40). At each visit, the patients filled a standardized coagulation questionnaire, addressing the usage of coagulation-altering medication, known coagulation/fibrinolysis disorders and any symptoms of potential coagulation disorders. In addition, their blood group was determined once after inclusion into the study

This prospective two-armed single center study was approved by the ethics committee, University of Erlangen (approval #82_19B, 20.03.2019). The recruitment period was from April 2019 to March 2021. Patients with advanced or metastatic skin cancer (melanoma *n* = 29; squamous cell carcinoma *n* = 1; Merkel cell carcinoma *n* = 1) were recruited at the skin cancer center Erlangen, Germany, before initiation of systemic tumor therapy with either ICI or BRAF/MEKi. Patients who had previously received ICI or BRAF/MEKi, with known coagulation–fibrinolysis disorders or under coagulation altering substances were excluded. All included patients gave written informed consent. The patients were treated according to standard clinical care with routine protocols according to the prescribing information. Two cohorts were compared based on the treatment they received (Fig. 1). Patients filled out a standardized coagulation questionnaire and were questioned for coagulation-altering medications. They were subsequently examined clinically and longitudinal blood samples were taken at each visit (baseline, day 7, 20 and 40; Fig. 2).

### Methods

Longitudinal blood samples were taken at baseline (t0) and on days 7, 20 and 40 after initiation of systemic tumor therapy (t7, t20 and t40). Approximately 40ml (24.5ml citrate blood, 4ml EDTA-anticoagulated blood, 3.8ml citrate buffered blood and 7.5ml serum) were taken at each time point to investigate coagulation parameters. Blood samples of patients, who took coagulation-altering medication at one or more sampling times after consenting to the study were excluded from the analysis. The samples were analyzed following standardized and validated procedures [28, 29]. The coagulation parameters were determined using Stago automate analyzer (STA R MAX 3, clotting or immunoturbidimetric tests), the hematological parameters were determined using a Sysmex automate analyzer (Sysmex XN2000). In order to additionally test for tissue factor levels the protocol was amended to include an additional 4.9ml of citrate blood for analysis [11]. Tissue factor concentrations were quantified with the Biolegend LEGENDPlex Human Thrombosis Panel in accordance to the manufacturer’s instructions. Blood samples were centrifuged for 10 min at 2500 *g*, aliquoted and stored at − 80°C for batch analysis. Samples were analyzed in the Translational Research Center Erlangen (TRC) with analyte concentrations being calculated using the LEGENDplex v8.0 software (VigeneTech, Carlisle, MA, USA). The additional blood was also used for a factor X activity analysis.

In total, 98 different blood parameters were assessed. These included blood count (erythrocytes, leukocytes, thrombocytes, hemoglobin, hematocrit, mean corpuscular hemoglobin (MCH), mean corpuscular volume (MCV), mean corpuscular hemoglobin concentration (MCHC), erythrocyte distribution, platelet-large cell ratio (P-LCR), normoblasts, mean platelet volume, proportion of microcytic erythrocytes, relative and absolute counts of granulocytes, immature granulocytes (IG), lymphocytes, basophils, eosinophils and monocytes), blood type (determined once), prothrombin time, International Normalized Ratio (INR), activated Partial Thromboplastin Time (aPTT), thrombin clotting time, fibrinogen, protein C, protein S, antithrombin, D-dimer, Lupus anticoagulant, anti-cardiolipin antibodies, beta2-glycoprotein-antibodies, factor VIII:C, von Willebrand factor antigen, von Willebrand factor activity, von Willebrand multimer analysis, Born aggregometry, platelet function analysis, Rotational thromboelastometry (ROTEM), interleukin 6 (IL-6), P-selectin, P-selectin glycoprotein ligand 1 (PSGL-1), tPA, sCD40L, PAI-1, tissue factor, factor IX, interleukin 8 (IL-8) and factor X activity.

The questionnaire filled out by the patients was a standardized questionnaire for coagulation history used at the University Hospital Erlangen [30]. In this survey, general risk factors for vascular diseases, known disorders of the coagulation/fibrinolysis system or the previous occurrence of unspecific symptoms of thromboses or bleeding events as well as medication intake and the family disposition for coagulation disorders were assessed (supplementary Table 1).

Data were organized in an electronic database. For analysis, all patients were included as intent-to-treat. The gathered data for each of the four sampling time points were pooled for both cohorts and analysis were performed using the mean values. Longitudinal changes induced by induction of treatment and differences between both cohorts were investigated and statistically analyzed via mixed-effects analysis with Geisser–Greenhouse correction and Tukey’s test for multiple comparisons. For patients undergoing adjuvant therapy recurrence-free survival (RFS) and overall survival (OS) were analyzed including calculation of univariate Cox proportional hazard regression analysis. For patients in the metastatic setting PFS and OS were calculated.

RFS or PFS and OS of patients with baseline low levels of coagulation parameters were compared to patients with high baseline coagulation parameters using Kaplan–Meier curves and log-rank (Mantel-Cox) tests with the median value being the cutoff. The adjuvant cohort was expanded with 6 additional patients undergoing ICI therapy to analyze the association of factor VIII:C and vWF ag and RFS. Due to the small cohort size of BRAF/MEK patients (4 adjuvant and 3 with metastatic cancer), no hazard analysis of baseline values of coagulation parameters for BRAF/MEK patients was performed.

*P-*values ≤ 0.05 were considered statistically significant. Graphs were produced via GraphPad Prism versions v8.3.0-v.10.3.1 (GraphPad Software, Boston, Massachusetts, USA), charts via Paint.NET v4.3.12 (dotPDN LLC, Seattle, WA, USA).

## Results

In total, 31 patients with advanced or metastatic skin cancer were included before initiation of systemic tumor therapy. Patients were treated with either ICIs (*n* = 24) or BRAF/MEKi (*n* = 7). The average age at inclusion was 62.5 years (range, 25–82 years). For patient characteristics, see Table 1. During the study period one patient stopped the systemic tumor therapy because of non-hematological side effects; another patient withdrew from the study. The gathered data were included up to that time point. During the observation period and follow-up, no thromboembolic events occurred. The average follow-up was 27.6 months. An overview of the main effects of both therapy regimens is presented in Table 2, the raw data can be found in supplementary Table 2 and supplementary Table 3.

**Table 1:** Patient characteristics

	Patients	
Cohort	Immune checkpoint inhibitor therapy (ICI)	BRAF/MEK inhibitor therapy (BRAF/MEKi)
Patients *n* (%)	24 (77.4%)	7 (22.6%)
Age (years) median (range)	63.6 (25–82 years)	58.4 (40–81 years)
Gender *n* (%)		
Male	8 (33.3%)	4 (57.1%)
Female	16 (66.7%)	3 (42.9%)
Type of cancer		
Melanoma	22 (91.6%)	7 (100%)
Cutaneous squamous cell carcinoma	1 (4.2%)	
Merkel cell carcinoma	1 (4.2%)	
Stage AJCC (2016) *n* (%)		
IIIB	7 (29.2%)	1 (14.2%)
IIIC	7 (29.2%)	3 (42.9%)
IV	10 (41.6%)	3 (42.9%)
Metastatic classification *n* (%)		
M0	14 (58.3%)	4 (57.1%)
M1	1 (4.2%)	0 (0%)
M1a	1 (4.2%)	1 (14.3%)
M1b	1 (4.2%)	0 (0%)
M1c	5 (20.8%)	0 (0%)
M1d	2 (8.3%)	2 (28.6%)
LDH *n* (%)		
Normal	11 (45.8%)	3 (42.9%)
Elevated above ULN	13 (54.2%)	4 (57.1%)
Therapy regime with interval *n* (%)		
Pembrolizumab (q3w)	11 (45.8%)	
Nivolumab (q2w)	7 (29.1%)	
Avelumab (q2w)	1 (4.2%)	
Cemiplimab (q3w)	1 (4.2%)	
Ipilimumab + Nivolumab (q3w)	4 (16.7%)	
Dabrafenib + Trametinib (daily)		7 (100%)
Primary therapy response *n* (%)		
Progression-free survival	12 (50%)	4 (57.1%)
Complete remission	2 (8.3%)	0 (0%)
Partial response	1 (4.2%)	2 (28.6%)
Stable disease	1 (4.2%)	0 (0%)
Progressive disease	8 (33.3%)	1 (14.2%)
Survival in months (*n* =)		
Patients with metastatic melanoma	PFS 15.5 (*n* = 8) OS 20.4 (*n* = 8)	PFS 14.1 (*n* = 3) OS 28.3 (*n* = 3)
Patients with adjuvant therapy	RFS 25.4 (*n* = 14) OS 31.2 (*n* = 14)	RFS 17.9 (*n* = 4) OS 33.8 (*n* = 4)

**Table 2:** Overview of the main effects of ICI and BRAF/MEKi on coagulation parameters

	Immune checkpoint inhibitors	BRAF/MEK inhibitors
Factor VIII:C	↑	(↗)
vWF activity	(↗)	(↗)
Antithrombin activity	(↘)	( →)
Protein S activity	↓	↓
Tissue factor	(↗)	n.a

*Significant changes under therapy (*p-*values ≤ 0.05) are shown as upward or downward-pointing, non-significant changes as diagonal arrows in brackets. Horizontal arrows in brackets indicate a constant course under therapy

**Fig. 3:**
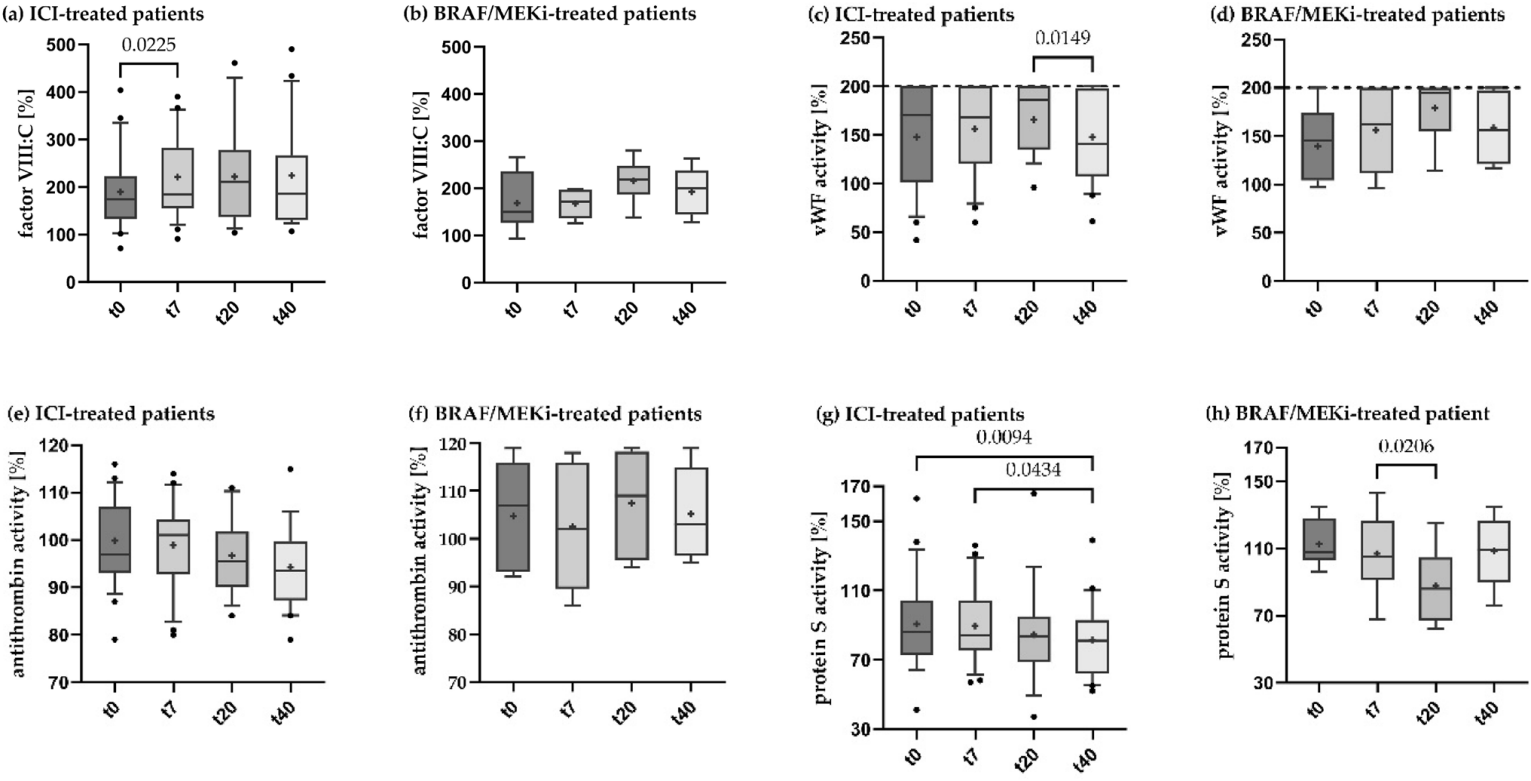
Changes in coagulation parameters under ICI and BRAF/MEKi therapy Longitudinal coagulation parameter values measured in the blood samples are shown as a box-and-whisker plot for baseline and days 7, 20 and 40 after initiation of therapy. + sign indicates mean value, horizontal bar the median, box shows interquartile range, whiskers the 10–90 percentile range, outliers are shown in form of dots. Statistical comparison of mean values using mixed-effects analysis, correction for multiple comparisons was performed using Tukey's correction, *p*-values ≤ 0.05 are displayed above brackets. t0 = baseline, t7, t20, t40 = days after initiation of systemic therapy. *n*(ICI) = 21–24; *n*(BRAF/MEKi) = 7

A significant increase in the mean values of factor VIII activity was seen (Fig. 3a) on day seven after initiation of ICI therapy compared to baseline, from 189.7 to 221.1% activity compared to the norm (*p*: 0.0225). This increased activity was measurable during the whole study period. A similar change could be observed in the BRAF/MEKi cohort; however, here the increased factor VIII activity occurred after three weeks and was more accentuated from 167.5 at t7 to 215.8% at t20 (*p*: 0.0587, Fig. 3b).

The measurements of the von Willebrand antigen (vWF ag) and the von Willebrand factor activity (vWF-ac) were limited due to the restricted analysis area used for standard analysis capping at 420% for the antigen and 200% for the factor activity. Values above the cutoff were included as 420% and 200%. Although statistically not significant, both, antigen and activity increased in the first 20 days after initiation of systemic therapy (Fig. 3c + d). After the initial increase, a significant decrease in the von Willebrand factor activity could be measured between t20 and t40 (*p:* 0.0149). Both parameters had a similar progression in the BRAF/MEKi-treated group, even if they were not statistically significant.

The parameters vWF-ac and factor VIII:C were significantly different at t7 in the group of ICI + CTLA-4 patients compared to the patients receiving Pembrolizumab monotherapy. For the other reported parameters there were no significant differences between the patient cohorts with different ICI treatment regimens at t7.

The antithrombin activity in the ICI group (Fig. 3e) decreased constantly during the observation period from 99.8% at therapy initiation to 94.3% after 40 days (*p:* 0.1083). This effect was not measurable in the BRAF/MEKi group to the same extend (Fig. 3f); however, differences between both groups were not significant.

We found a constant reduction in the protein S activity in both cohorts (Fig. 3g) from 90.5% at t0 and 89.7% at t7 to 81.6% at t40 in the ICI group and a similar reduction in the control group treated with BRAF/MEKi between the sampling times 7 and 20 days after therapy initiation (Fig. 3h). We found no significant differences between both cohorts.

Furthermore, notable effects of ICI on the coagulation in the rotational thromboelastometry (ROTEM) were found. In the intrinsic thromboelastometry (INTEM), a subtest within the ROTEM-analysis to assess the clotting factors of the intrinsic system, covering platelets and fibrinolysis, revealed that the clot formation time (INTEM-CFT) rose in the first week from an average 56.3s to 60.9s at t7. The clot formation time (CFT) normalized (55,0s at t20) in the following two weeks and sunk even further to an average of 53.1s at t40 (*p*: 0.0149). This impact could not be measured in the cohort treated with BRAF/MEKi, where a CFT reduction in the first week of therapy and a normalization afterward was observed. In the FIBTEM analysis of the ICI group, where functional fibrinogen levels and fibrin polymerization can be measured by suppressing the platelet activity via cytochalasin D a reduced clot formation time could be measured at t20 (*p:* 0.0147) after an initial increase between initiation and t7.

**Fig. 4:**
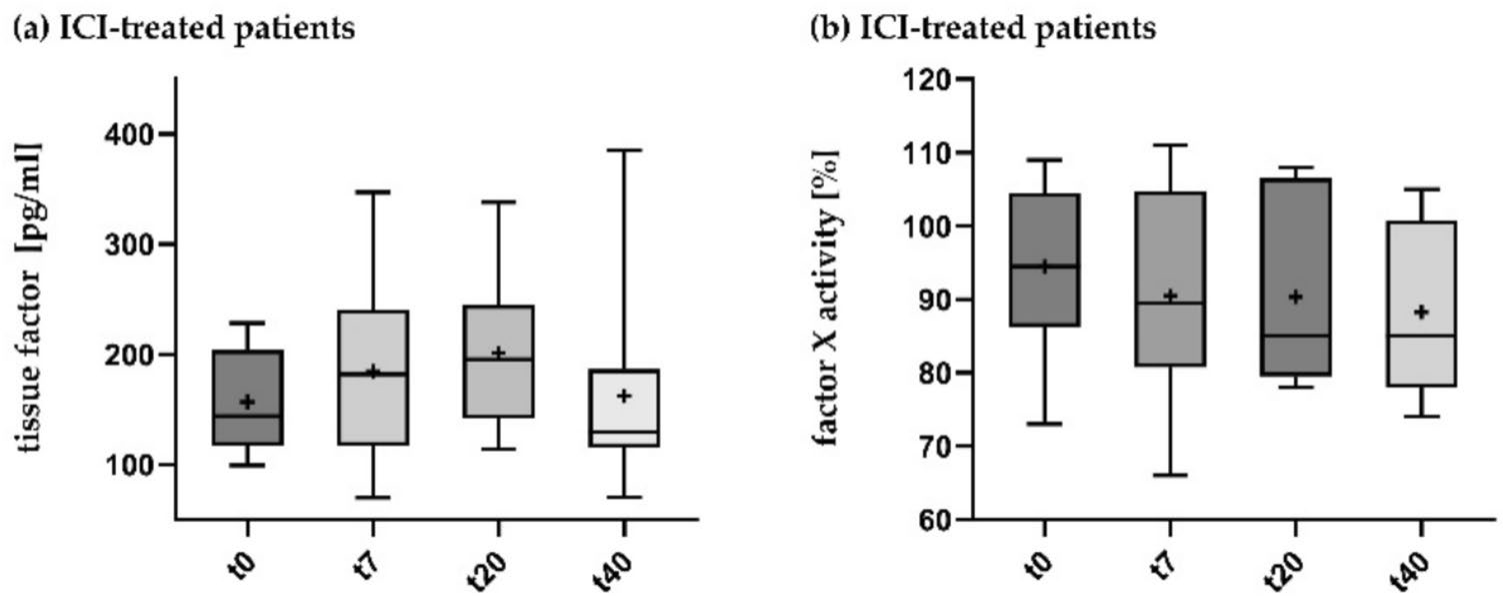
Changes in tissue factor concentration and factor X activity under ICI therapy The tissue factor concentration and factor X activity measured in blood samples shown as box-and-whisker plot for longitudinal time points after initiation of ICI therapy. Horizontal bar indicates the median, box shows interquartile range, whiskers the 10–90 percentile range. Statistical comparison of mean values performed using mixed-effects analysis, correction for multiple comparisons was performed using Tukey's correction t0 = baseline, t7, t20, t40 = days after initiation of systemic therapy. *n*(ICI) = 8

The tissue factor concentration in the blood was analyzed from eight ICI patients using the Biolegend LEGENDPlex Human Thrombosis Panel (Fig. 4a). Here a steady increase in the tissue factor concentration was measurable in the first three weeks of therapy (t0: 156.6 ng/ml; t20: 201.2 ng/ml) although not being significant (*p:* 0.1847). Similarly, increasing concentrations of IL-6 and PSGL-1 were measurable under ICI therapy.

Factor X activity was also determined from eight ICI patients (Fig. 4b). Apart from a single value measured with reduced activity, all measurements were in the normal range. In the statistical evaluation, there were no significant changes in factor X activity under ICI therapy.

To determine the influence of the investigated coagulation parameters on the progression-free (PFS) or recurrence-free (RFS) and overall survival (OS) under ICI therapy, patients were analyzed using univariate Cox proportional hazard regression. For the hazard analysis of baseline vWF ag and factor VIII:C on RFS and OS the cohort was complemented with 6 melanoma patients before start of adjuvant therapy (55–83 years of age, mean 71.8 years).

As in the prospective study cohort, increased baseline levels of factor VIII:C (HR 1.050; confidence interval (CI) 1.019 to 1.096; p: 0.0065) and vWF ag (HR 1.031; CI 1.006 to 1.062; p: 0.0251) were also associated with an increased risk of cancer recurrence in the expanded adjuvant patient cohort (*n* = 20). The hazard ratios in the group with metastatic melanoma (*n* = 8) were non-significant with 1.004 for factor VIII:C (*p:* 0.3495) and 1.005 for vWF ag (*p:* 0.1840). No statistically significant worse PFS/RFS could be detected for patients with reduced baseline antithrombin activity (adjuvant: HR 0.9133; CI 0.8137 to 1.007; *p:* 0.0841; metastatic: HR 0.9914; CI 0.8961 to 1.114; *p:* 0.8740) and vWF activity (adjuvant: HR 1.027; CI 1.004 to 1.070; *p:* 0.0709; metastatic: HR 1.009; CI 0.9944 to 1.027; *p:* 0.2628) in both cohorts.

There were no significant correlations between the baseline values of vWF ag, vWF-ac, factor VIII:C and antithrombin activity and the OS.

To analyze the clinical relevance of these hazard ratios, the cohort treated with ICI in an adjuvant setting was divided into two subgroups (low baseline blood parameter levels compared to high baseline blood parameter levels) using the median. The RFS and OS of both groups were then compared using Kaplan–Meier curves and log-rank (Mantel-Cox) tests.

**Fig. 5:**
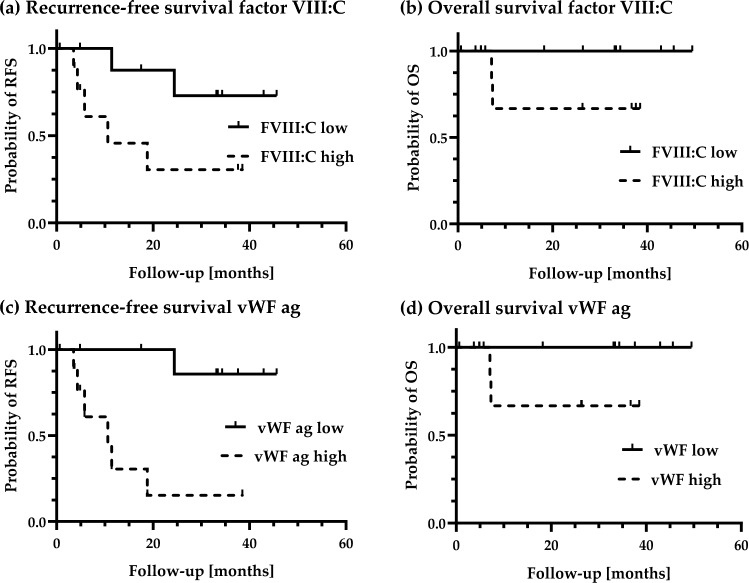
Effect of baseline levels of factor VIII:C and vWF ag on RFS and OS in patients treated in an adjuvant setting with ICI Patients treated in the adjuvant setting with ICI (*n* = 20) were divided into two subgroups based on the median baseline levels of factor VIII:C (175%) and vWF ag (127%), respectively. The recurrence-free and overall survival was then plotted via Kaplan–Meier curves with either low (solid line) or high baseline parameter values (dotted line). Cancer recurrence or deaths are displayed as a step in the curve, patients that were lost to follow-up, had no recurrence or were alive by the end of the observation period were censored in analysis and marked with a vertical line in the figure. Recurrence-free and overall survival was statistically analyzed via log-rank (Mantel-Cox) test. There was a significant difference in recurrence-free (p(factor VIII:C)*:* 0.047; p(vWF ag): 0.002) but not for overall survival between cohorts for both factor VIII:C and vWF ag (*p:* 0.0872). *n*(parameter low) = 10; *n*(parameter high) = 10

Here we found a 4.5 times higher risk (CI 0.9535 to 20.93; *p:* 0.047) for cancer recurrence in patients if the baseline value of factor VIII:C was above the median of 175% (Fig. 5a) and a 12.5 times higher risk (CI 2.562 to 60.58; *p:* 0.002) if the baseline vWF ag was above the median of 127% (Fig. 5c). There were no significant differences in overall survival between both groups for factor VIII:C and vWF ag (*p:* 0.0872) (Fig. 5b + d).

## Discussion

This prospective clinical study provides evidence that ICI or BRAF/MEKi therapy induce a pro-coagulant state in skin cancer patients. It further implies that increased baseline levels of von Willebrand factor antigen and factor VIII:C before initiation of ICI therapy correlates with a significantly higher risk for recurrence in melanoma patients treated in the adjuvant setting.

These results are highly relevant in view of the controversial role of ICI therapy in the development of thromboembolic events [10, 12, 24, 25], especially since cancer patients already have a 4–9 fold increased risk of thromboembolic events [16, 17] due to their increased coagulation activity.

Based on meta-analyses showing an incidence of 1.5–2.7% for VTEs and 1.1% for ATEs [24, 31] under ICI therapy, it is still unclear whether patients treated with ICI therapy have an even further increased risk of thromboembolic events compared to non-ICI-treated patients. However, underreporting of thromboembolic events in these critically ill patients developing various irAEs is possible [26]. BRAF/MEKi therapy has already been associated with venous and pulmonary thromboembolism [14].

The elevated risk for thromboembolic events (TEE) in cancer in general is due to an increased coagulation activity by various mechanisms like the release of coagulation-stimulating factors and activators such as tissue factor or coagulation factors like factor VII and factor X as well as inflammatory cytokines by the cancer cells, which can lead to endothelial damage, dysfunction and induction of the coagulation cascade and platelet activity—as well as fibrinolysis-inhibiting substances such as plasminogen activator inhibitor 1 (PAI-1) [18].

The ICI-induced risk for TEE is thought to be associated with an early flare-up of C-reactive protein (CRP) [32]. This flare-up has already been confirmed as a predictive marker for better treatment response under ICIs [33] and an increased risk for the occurrence of immune-related side effects [34, 35].

Since the median onset of hem-irAEs was 12 weeks for patients with combinational ICI therapy and 25 weeks for ICI monotherapy [11] and thromboembolic events are rare, it is not surprising that no events were observed in this study. In addition, unfortunately, the two cohorts had a skewed gender distribution with a higher share of women. This could bias the results since females have a lower age-adjusted incidence of VTEs [36]. Since patients had no direct benefit but study participation required additional visits for blood draws only a very low percentage agreed to participate. Although the cohorts were small with a resulting susceptibility to outliers, the rigorous longitudinal assessments of coagulation parameters could show an increase in factor VIII:C and vWF activity and a reduction of protein S activity within both ICI and BRAF/MEKi-treated cohorts. In addition, a significant reduction in the activity of coagulation-inhibiting antithrombin was observed in the ICI-treated patient cohort only.

Previous research has shown a correlation between increased levels of coagulation factors and higher relative risks of venous thrombosis [37, 38]. Of these, increased levels of factor VIII:C and the von Willebrand factor were found to be most strongly associated to higher risks of venous thrombosis [39]. Increased levels of these under ICI therapy, as detected in the presented data, could be amplifiers in the occurrence of coagulation–fibrinolysis disorders. The already elevated activity levels at therapy initiation could have been caused by the presence of a malignant tumor disease [40]. Additional to the role of factor VIII in the coagulation cascade, high levels of factor VIII are likely to induce a resistance to the anticoagulant effects of activated protein C (APC) and its cofactor protein S [38]. According to that, reductions in both protein C and protein S activity were demonstrated of which, however, only the reduction of protein S was significant in both cohorts.

Importantly, the induction of a pro-coagulant state was shown in patients treated with either ICI or BRAF/MEKi. Due to the small patient cohorts presented in this study, the highly relevant prognostic significance of factor VIII:C and vWF ag in the adjuvant ICI therapy needs to be confirmed in larger clinical trials.

In contrast to the data presented, a significantly increased risk of tumor progression and a poorer response during ICI therapy in metastatic patients with higher levels of vWF ag was observed in another prospective study [41]. The correlation could be due to the close reciprocal linkage between immune- and hemostatic system. As an example for this close connection, platelets have been shown to promote cancer metastasis through downregulation of natural killer cell response to circulating cancer cells and by attaching to tumor cells and promoting extravasation [42]. Since platelet activation plays an important role in cancer-associated thrombosis and tumor immune evasion a previous study investigated direct effects of ICI on platelets, however found no clear effect [43].

Based on an improved understanding of the influence of cancer on the immune and coagulation systems and vice versa, studies have already demonstrated a positive effect on cancer therapy by influencing the coagulation system. For example, myeloid cells of the tumor microenvironment (TME) secrete the coagulation factor Xa, which acts as a regulator of immune cell activation and contributes to tumor immune evasion [44], and factor Xa inhibitor (rivaroxaban) usage increases the anti-tumor response of mice to ICI therapy. This synergistic effect of ICI and factor Xa inhibitors was also demonstrated in cancer patients where a co-medication with rivaroxaban improved therapy response; while, the risk of bleeding remained unchanged [45]. However, the analysis of blood samples from eight ICI-treated patients included in the present study did not show increased factor X activity at baseline or significant changes of factor x activity during ICI therapy.

In conclusion, systemic anti-tumor therapy with ICI or BRAF/MEKi induces an increased expression and release of pro-coagulant factors. This, paired with reduced activity of coagulation-inhibiting factors, leads to hemostatic imbalances in patients treated with these therapeutic regimens and a subsequently further increased risk of thromboembolic events. Increased levels of pro-coagulant factors such as vWF ag or factor VIII:C correlated with an increased risk of cancer recurrence in melanoma patients treated with ICI in the adjuvant setting. A better understanding of this interplay could enable therapeutic targeting of the coagulative system to improve patient outcome.

## Supplementary Information

Below is the link to the electronic supplementary material.

Supplementary file1 (DOCX 124 KB).

## Data Availability

Data are contained within the article or supplementary material.

## Abbreviations

ATE: Arterial thromboembolism
APC: Activated protein C
AJCC: American Joint Committee on Cancer
aPTT: Activated thromboplastin time
BRAF: Proto-oncogene B-Raf
BRAF/MEKi: BRAF/MEK inhibitor therapy
CFT: Clot formation time
CI: Confidence interval
CRP: C-reactive protein
EDTA: Ethylenediaminetetraacetate
FIBTEM: Fibrin thromboelastometry
HR: Hazard ratio
hem-irAEs: Hematological immune-related adverse events
ICI: Immune checkpoint inhibitors
IG: Immature granulocytes
IL-6: Interleukin-6
IL-8: Interleukin 8
IL-10: Interleukin-10
IL-1β: Interleukin-1β
IL-17: Interleukin-17
INR: International normalized ratio
INTEM: Intrinsic thromboelastometry
INTEM CFT: Intrinsic thromboelastometry-clot formation time
irAE: Immune-related adverse events
LDH: Lactate dehydrogenase
MCH: Mean corpuscular hemoglobin
MCV: Mean corpuscular volume
MCHC: Mean corpuscular hemoglobin concentration
MEK: Mitogen-activated protein kinase kinase
NSCLC: Non-small-cell lung cancer
OS: Overall survival
PAI-1: Plasminogen activator inhibitor-1
PD-1: Programmed cell death protein 1
PD-L1: Programmed cell death-ligand 1
P-LCR: Platelet-large cell ratio
PSGL-1: P-selectin glycoprotein ligand 1
PFS: Progression-free survival
ROR: Reported odds ratio
ROTEM: Rotational thromboelastometry
RFS: Recurrence-free survival
sCD40L: Soluble CD40 ligand
TEE: Thromboembolic event
TF: Tissue factor
TME: Tumor microenvironment
TRC: Translational research center
tPA: Tissue plasminogen activator
ULN: Upper limit of normal
VTE: Venous thromboembolism
vWF: Von Willebrand factor
vWF ag: Von Willebrand factor antigen
vWF-ac: Von Willebrand factor activity
