# Supplementary material for: Interdependence of coagulation with immunotherapy and BRAF/MEK inhibitor therapy: results from a prospective study
Source: Cancer Immunol Immunother. 2024 Nov 2;74(1):5. doi: 10.1007/s00262-024-03850-y (PMC11531462; doi:10.1007/s00262-024-03850-y)
Supplement: Supplementary file 1 — Supplementary file1 (DOCX 124 KB). [file 262_2024_3850_MOESM1_ESM.docx]

**SUPPLEMENTARY MATERIAL**

**Supplementary Table 1: Special coagulation history questionnaire**

**Special coagulation history questionnaire**

Patient label:

Body weight : __________ kg Body size: ____________ cm

**General risk factors for vascular disease:**

Do you have high blood pressure? ☐ yes ☐ no

Do you have diabetes? ☐ yes ☐ no

Do you smoke? ☐ yes ☐ no

If yes, how many per day? …………………………………………………….

Do you have elevated blood lipids? ☐ yes ☐ no

Have you had any operations? ☐ yes ☐ no

If yes, which ones and when? ……………………………………………………..

Do you have any known heart conditions? ☐ yes ☐ no

If yes, which ones? ……………………………………………………..

**Bleeding history:**

Have you had mucosal bleeding (epistaxis, gingival bleeding, menorrhagia)? ☐ yes ☐ no

Have you had any joint bleeding? ☐ yes ☐ no

Have you experienced increased nasal bleeding in yourself, even for no apparent reason? ☐ yes ☐ no

Do you have or have you had more "bruises" or small punctiform bleedings without bumping yourself? ☐ yes ☐ no

Have you experienced bleeding of the gums for no apparent reason? ☐ yes ☐ no

Did you experience prolonged and increased bleeding after tooth extraction? ☐ yes ☐ no

Do you have the impression that cuts or abrasions (e.g. shaving) bleed for a longer period of time?

☐ yes ☐ no

Have you ever experienced prolonged and increased bleeding after or during surgery (e.g. tonsillectomy, appendectomy, childbirth, etc.)? ☐ yes ☐ no

Have you ever been given blood units or blood products during an operation? ☐ yes ☐ no

Please indicate the type of surgery: ………………………….

Does bleeding or "bruising" occur after minor bumps/injuries, so-called minor trauma

more than 1 to 2 times per week ☐ yes ☐ no

or 1 to 2 times per week ☐ yes ☐ no

Or 1 to 2 times per month? ☐ yes ☐ no

For women/girls: Do you have the impression that your menstrual bleeding is prolonged (>7 days) and/or increased (frequent tampon changes)? ☐ yes ☐ no

**Thrombosis and anticoagulants:**

Have you had thrombosis of any kind (leg vein thrombosis, etc.)? ☐ yes ☐ no

If yes, when and at what site (leg, pelvis, etc.): ………………………….

Do you have or have you had pulmonary embolism(s)? ☐ yes ☐ no

For women: Are you taking birth control pills (oral contraceptives)? ☐ yes ☐ no

Are you taking Marcumar or similar anticoagulants? ☐ yes ☐ no

If yes, which ones? ………………………….

Are you taking or have you taken ASA (aspirin, etc.) in the past few days? ☐ yes ☐ no

Are you taking any pain or anti-rheumatic medications? ☐ yes ☐ no

If yes, which ones? ………………………….

Are you taking any other medications? ☐ yes ☐ no

If yes, which ones? ………………………….

**Family history:**

Are you aware of a case of bleeding tendency

-in your grandparents ☐ yes ☐ no

-in your parents ☐ yes ☐ no

-in your children ☐ yes ☐ no

Are you aware of a case of venous thrombosis or embolism

- in your grandparents ☐ yes ☐ no

- in your parents ☐ yes ☐ no

-in your children ☐ yes ☐ no

If yes, what / when / with whom? ………………………….

…………………………………………………………………………......................

. . . . . . . . . . . . . . .. . . . . . . . . . . . . . . . . . . . . . . . . . . . . . . .

Date Signature of patient Doctor's signature

The questionnaire presented is an english translation of the german questionnaire used in the study

**Supplementary Table 2: Measured mean values under ICI therapy**

|  | **t0 (n)** | **t7 (n)** | **t20 (n)** | **t40 (n)** | **Comparison** | **Mean Diff.** | **95% CI of diff.** | **Adj. *p*-values** | **Geisser-Greenhouse’s epsilon** |
| --- | --- | --- | --- | --- | --- | --- | --- | --- | --- |
| Factor VIII:C [%} | 189.7  (n= 24) | 221.1  (n= 22) | 221.7  (n= 19) | 224.5  (n= 21) | t0 vs. t7 t0 vs. t20  t0 vs. t40  t7 vs. t20 t7 vs. t40 t20 vs. t40 | -31.38 -32.03 -34.77 -0.6459 -3.385 -2.739 | -59.04 to -3.724 -90.28 to 26.22 -99.49 to 29.95 -53.53 to 52.24 -49.62 to 42.85 -59.93 to 54.46 | **0.0225** 0.4280  0.4539  >0.9999 0.9967  0.9991 | 0.7190 |
| vWF antigen [%] | 151.5  (n= 24) | 172.1  (n= 22) | 180.7  (n= 19) | 179.9  (n= 21) | t0 vs. t7 t0 vs. t20  t0 vs. t40  t7 vs. t20 t7 vs. t40 t20 vs. t40 | -20.55  -29.20 -28.32 -8.650 -7.770 0.8797 | -45.62 to 4.527 -79.20 to 20.81 -86.97 to 30.34 -63.42 to 46.12 -64.81 to 49.27 -45.81 to 47.57 | 0.1346 0.3773  0.5428  0.9689 0.9803  >0.9999 | 0.6145 |
| vWF activity [%] | 147.7  (n= 24) | 156.0  (n= 22) | 165.8  (n= 19) | 148.1  (n= 21) | t0 vs. t7 t0 vs. t20  t0 vs. t40  t7 vs. t20 t7 vs. t40 t20 vs. t40 | -8.288 -18.18 -0.4762 -9.888 7.812 17.70 | -29.11 to 12.53 -44.26 to 7.913 -26.67 to 25.72 -30.59 to 10.82 -15.32 to 30.95 3.088 to 32.31 | 0.6878 0.2360 >0.9999 0.5368 0.7764 **0.0149** | 0.7845 |
| Antithrombin activity [%] | 99.83  (n= 23) | 98.95  (n= 22) | 96.69  (n= 16) | 94.30  (n= 20) | t0 vs. t7 t0 vs. t20  t0 vs. t40  t7 vs. t20 t7 vs. t40 t20 vs. t40 | 0.8715 3.139 5.526 2.267 4.655 2.388 | -5.432 to 7.175 -3.177 to 9.454 -0.9201 to 11.97 -3.866 to 8.401 -0.7448 to 10.05 -3.203 to 7.978 | 0.9798 0.4997 0.1083 0.7044 0.1049 0.6060 | 0.8790 |
| Protein S activity [%] | 90.48  (n= 21) | 89.73  (n= 22) | 84.44  (n= 18) | 81.57  (n= 21) | t0 vs. t7 t0 vs. t20  t0 vs. t40  t7 vs. t20 t7 vs. t40 t20 vs. t40 | 0.7489 6.032 8.905 5.283 8.156 2.873 | -6.659 to 8.157 -2.212 to 14.28 1.993 to 15.82 -5.638 to 16.20 0.1986 to 16.11 -5.647 to 11.39 | 0.9916 0.1972 **0.0094** 0.5216 **0.0434** 0.7709 | 0.5470 |
| INTEM Clot formation Time [sec.] | 56.33  (n= 24) | 60.95  (n= 20) | 55.05  (n= 19) | 53.10  (n= 21) | t0 vs. t7 t0 vs. t20  t0 vs. t40  t7 vs. t20 t7 vs. t40 t20 vs. t40 | -4.617 1.281 3.238 5.897 7.855 1.957 | -11.27 to 2.042 -10.82 to 13.38 -3.713 to 10.19 -0.6043 to 12.40 1.371 to 14.34 -8.488 to 12.40 | 0.2415 0.9904 0.5711 0.0824 **0.0149** 0.9499 | 0.4896 |
| FIBTEM Clot formation time [sec.] | 392.7  (n= 15) | 467.3  (n= 15) | 142.9  (n= 14) | 364.5  (n= 13) | t0 vs. t7 t0 vs. t20  t0 vs. t40  t7 vs. t20 t7 vs. t40 t20 vs. t40 | -74.67 249.7 28.21 324.4 102.9 -221.5 | -162.2 to 12.91 52.00 to 447.5 -205.4 to 261.8 23.84 to 625.0 -215.4 to 421.2 -605.3 to 162.2 | 0.1034 **0.0147** 0.9806 **0.0345** 0.7485 0.3514 | 0.5376 |
| Tissue factor [pg/ml] | 156.6  (n= 8) | 184.4  (n= 8) | 201.2  (n= 6) | 162.0  (n= 8) | t0 vs. t7 t0 vs. t20  t0 vs. t40  t7 vs. t20 t7 vs. t40 t20 vs. t40 | -27.73  -44.52  -5.324  -16.79  22.41  39.20 | -102.5 to 47.02  -111.7 to 22.61  -94.74 to 84.09  -104.6 to 71.03  -34.67 to 79.49  -31.09 to 109.5 | 0.6304  0.1847  0.9970  0.8909  0.5912  0.2823 | 0.3641 |
| IL-6 [pg/ml] | 63.25  (n= 8) | 84.95  (n= 8) | 83.42  (n= 6) | 61.67  (n= 8) | t0 vs. t7 t0 vs. t20  t0 vs. t40  t7 vs. t20 t7 vs. t40 t20 vs. t40 | -21.70  -20.17  1.575  1.533  23.28  21.74 | -71.31 to 27.91  -57.38 to 17.04  -31.65 to 34.80  -38.37 to 41.44  -6.205 to 52.76  4.514 to 38.97 | 0.5118  0.3004  0.9985  0.9988  0.1244  **0.0203** | 0.04011 |
| PSGL-1 [pg/ml] | 846.1  (n= 8) | 966.7  (n= 8) | 935.0  (n= 6) | 994.9  (n= 8) | t0 vs. t7 t0 vs. t20  t0 vs. t40  t7 vs. t20 t7 vs. t40 t20 vs. t40 | -120.5  -88.81  -148.7  31.73  -28.19  -59.92 | -263.5 to 22.39  -366.5 to 188.9  -395.9 to 98.45  -148.7 to 212.2  -223.6 to 167.3  -136.7 to 16.81 | 0.0984  0.6631  0.2760  0.9116  0.9617  0.1153 | 0.6509 |
| Factor X activity [%] | 94.50  (n= 8) | 90.50  (n= 8) | 90.33  (n= 6) | 88.25  (n= 8) | t0 vs. t7 t0 vs. t20  t0 vs. t40  t7 vs. t20 t7 vs. t40 t20 vs. t40 | 4.0  4.167  6.250  0.1667  2.250  2.083 | -3.962 to 11.96  -6.494 to 14.83  -7.984 to 20.48  -8.523 to 8.857  -8.288 to 12.79  -5.467 to 9.633 | 0.4068  0.5287  0.5091  0.9999  0.8913  0.7474 | 0.2656 |
|  |  |  |  |  |  |  |  |  |  |
| Leukocyte count [10^3/µl] | 6.736  (n= 24) | 6.110  (n= 20) | 5.152  (n= 20) | 5.810  (n= 22) | t0 vs. t7 t0 vs. t20  t0 vs. t40  t7 vs. t20 t7 vs. t40 t20 vs. t40 | 0.6257 1.584 0.9257 0.9583 0.3000 -0.6583 | -0.2243 to 1.476 0.3259 to 2.842 -0.4818 to 2.333 -0.1973 to 2.114 -0.9882 to 1.588 -2.516 to 1.199 | 0.8719 0.5218 >0.9999 0.5267 0.7863 0.2811 | 0.5871 |
| Erythrocyte count [10^6/µl] | 4.513  (n= 24) | 4.484  (n= 21) | 4.356  (n= 20) | 4.513  (n= 22) | t0 vs. t7 t0 vs. t20  t0 vs. t40  t7 vs. t20 t7 vs. t40 t20 vs. t40 | 0.029 0.157 -0,0009  0.128 -0.029 -0,157 | -1.1665 to 0.2903 -0.1396 to 0.04116 -0.2671 to 0.2653 -0.1127 to 0.3261 -0.2730 to 0.1372 -0.4130 to 0.0856 | 0.8719  0.5218  >0.999  0.5267  0.7863  0.2811 | 0.8853 |
| Hemoglobin [g/dl] | 13.08  (n= 24) | 13.14  (n= 20) | 12.46  (n= 20) | 12.81  (n=22 ) | t0 vs. t7 t0 vs. t20  t0 vs. t40  t7 vs. t20 t7 vs. t40 t20 vs. t40 | -0.05667 0.6233 0.2697 0.6800 0.3264 -0.3536 | -0.6007 to 0.4874 -0.1316 to 1.378 -0.4720 to 1.011 0.02538 to 1.335 -0.1614 to 0.8141 -1.056 to 0.3491 | 0.9910 0.1282 0.7434 **0.0404** 0.2640 0.5023 | 0.8104 |
| Hematocrit [%] | 39.91  (n= 24) | 39.63  (n= 20) | 38.28  (n= 20) | 39.54  (n= 22) | t0 vs. t7 t0 vs. t20  t0 vs. t40  t7 vs. t20 t7 vs. t40 t20 vs. t40 | 0.2833 1.633 0.3674 1.350 0.08409 -1.265 | -2.031 to 2.598 -0.8275 to 4.093 -1.920 to 2.654 -0.7238 to 3.423 -1.379 to 1.547 -3.383 to 0.8526 | 0.9855 0.2751 0.9693 0.2823 0.9984 0.3580 | 0.7861 |
| Platelet count [10^3/µl] | 289.0  (n= 24) | 285.5  (n=)20 | 305.5  (n= 20) | 308.0  (n= 22) | t0 vs. t7 t0 vs. t20  t0 vs. t40  t7 vs. t20 t7 vs. t40 t20 vs. t40 | 3.592 -16.46 -19.00 -20.05 -22.60 -2.545 | -21.83 to 29.01 -53.16 to 20.24 -71.56 to 33.55 -46.23 to 6.132 -65.29 to 20.10 -51.11 to 46.02 | 0.9781 0.5976 0.7466 0.1680 0.4566 0.9988 | 0.5619 |
| MCH [pg] | 28.95  (n= 24) | 29.03  (n= 20) | 28.55  (n= 20) | 28.39  (n= 22) | t0 vs. t7 t0 vs. t20  t0 vs. t40  t7 vs. t20 t7 vs. t40 t20 vs. t40 | -0.07083 0.4042 0.5678 0.4750 0.6386 0.1636 | -0.4618 to 0.3201 -0.1824 to 0.9907 -0.2027 to 1.338 0.1567 to 0.7933 0.1589 to 1.118 -0.2460 to 0.5733 | 0.9558 0.2462 0.2008 **0.0030 0.0073** 0.6770 | 0.1861 |
| MCHC [g/dl] | 33.06  (n= 24) | 33.11  (n= 20) | 32.48  (n= 20) | 32.33  (n= 22) | t0 vs. t7 t0 vs. t20  t0 vs. t40  t7 vs. t20 t7 vs. t40 t20 vs. t40 | -0.05167 0.5833 0.7311 0.6350 0.7827 0.1477 | -0.3690 to 0.2657 0.08478 to 1.082 0.3214 to 1.141 0.1579 to 1.112 0.3726 to 1.193 -0.2109 to 0.5063 | 0.9673 **0.0185 0.0003 0.0076 0.0002** 0.6560 | 0.8667 |
| MCV [fl] | 87.62  (n= 24) | 87.62  (n= 20) | 87.96  (n= 20) | 87.77  (n= 22) | t0 vs. t7 t0 vs. t20  t0 vs. t40  t7 vs. t20 t7 vs. t40 t20 vs. t40 | 0.001667 -0.3433 -0.1561 -0.3450 -0.1577 0.1873 | -1.317 to 1.321 -2.052 to 1.365 -2.347 to 2.035 -1.298 to 0.6076 -1.353 to 1.037 -1.062 to 1.437 | >0.9999 0.9412 0.9971 0.7313 0.9814 0.9737 | 0.2025 |
| Erythrocyte distribution [%] | 14.04  (n= 24) | 13.74  (n= 20) | 14.89  (n= 20) | 14.91  (n= 22) | t0 vs. t7 t0 vs. t20  t0 vs. t40  t7 vs. t20 t7 vs. t40 t20 vs. t40 | 0.3025 -0.8525 -0.8716 -1.155 -1.174 -0.01909 | -0.0006094 to 0.6056 -2.106 to 0.4015 -1.688 to -0.05531 -2.009 to -0.3014 -1.953 to -0.3956 -0.7018 to 0.6636 | 0.0506 0.2564 **0.0336 0.0067 0.0025** 0.9998 | 0.2927 |
| Microcytic erythrocytes [%] | 2.758  (n= 24) | 2.259 (n= 20) | 3.290  (n= 20) | 3.309  (n= 22) | t0 vs. t7 t0 vs. t20  t0 vs. t40  t7 vs. t20 t7 vs. t40 t20 vs. t40 | 0.4995 -0.5317 -0.5508 -1.031 -1.050 -0.01909 | -0.2652 to 1.264 -1.263 to 0.1999 -1.744 to 0.6420 -1.761 to -0.3013 -1.999 to -0.1014 -1.147 to 1.109 | 0.2795 0.2075 0.5808 **0.0052 0.0278** >0.9999 | 0.2924 |
| Granulocyte count (absolute) [10^3/µl] | 5.103  (n= 24) | 5.070 (n= 20) | 5.533  (n= 20) | 5.493  (n= 22) | t0 vs. t7 t0 vs. t20  t0 vs. t40  t7 vs. t20 t7 vs. t40 t20 vs. t40 | 0.03292 -0.4296 -0.3898 -0.4625 -0.4227 0.03977 | -1.307 to 1.373 -2.277 to 1.417 -1.370 to 0.5904 -2.173 to 1.248 -1.693 to 0.8476 -2.034 to 2.114 | 0.9999 0.9128 0.6884 0.8652 0.7809 >0.9999 | 0.6562 |
| Granulocyte count (relative)  [%] | 65.08  (n= 24) | 64.60 (n= 20) | 67.23  (n= 20) | 68.43  (n= 22) | t0 vs. t7 t0 vs. t20  t0 vs. t40  t7 vs. t20 t7 vs. t40 t20 vs. t40 | 0.4833 -2.147 -3.344 -2.630 -3.827 -1.197 | -4.528 to 5.495 -5.809 to 1.516 -9.350 to 2.662 -7.655 to 2.395 -10.51 to 2.851 -6.796 to 4.402 | 0.9928 0.3770 0.4261 0.4617 0.3895 0.9294 | 0.7743 |
| Lymphocyte count (absolute) [10^3/µl] | 1.853  (n= 24) | 1.615  (n= 20) | 1.489  (n= 20) | 1.542  (n= 22) | t0 vs. t7 t0 vs. t20  t0 vs. t40  t7 vs. t20 t7 vs. t40 t20 vs. t40 | 0.2383 0.3643 0.3115 0.1260 0.07318 -0.05282 | 0.03056 to 0.4461 0.02770 to 0.7010 -0.2854 to 0.9084 -0.1091 to 0.3611 -0.5624 to 0.7088 -0.6129 to 0.5073 | **0.0212 0.0311** 0.4812 0.4418 0.9875 0.9931 | 0.4412 |
| Lymphocyte count (relative) [%] | 24.31  (n= 24) | 22.47 (n= 20) | 20.55  (n= 20) | 19.61  (n= 22) | t0 vs. t7 t0 vs. t20  t0 vs. t40  t7 vs. t20 t7 vs. t40 t20 vs. t40 | 1.848 3.768 4.703 1.920 2.856 0.9359 | -2.049 to 5.744 0.8682 to 6.667 -0.1617 to 9.568 -1.562 to 5.402 -2.543 to 8.255 -3.741 to 5.613 | 0.5540 **0.0084** 0.0605 0.4181 0.4570 0.9410 | 0.7740 |
| Eosinophil count (absolute) [10^3/µl] | 0.1863  (n= 24) | 0.2065 (n= 20) | 0.2205  (n= 20) | 0.2173  (n= 22) | t0 vs. t7 t0 vs. t20  t0 vs. t40  t7 vs. t20 t7 vs. t40 t20 vs. t40 | -0.02025 -0.03425 -0.03102 -0.01400 -0.01077 0.003227 | -0.1111 to 0.07063 -0.1025 to 0.03402 -0.1307 to 0.06868 -0.1125 to 0.08450 -0.1239 to 0.1024 -0.08781 to 0.09427 | 0.9223 0.5082 0.8215 0.9765 0.9928 0.9996 | 19.61 |
| Eosinophil count (relative) [%] | 2.396  (n= 24) | 2.825 (n= 20) | 3.170  (n= 20) | 3.177  (n= 22) | t0 vs. t7 t0 vs. t20  t0 vs. t40  t7 vs. t20 t7 vs. t40 t20 vs. t40 | -0.4292 -0.7742 -0.7814 -0.3450 -0.3523 -0.0072 | -1.543 to 0.6843 -1.809 to 0.2603 -2.074 to 0.5108 -1.579 to 0.8885 -1.975 to 1.271 -1.173 to 1.158 | 0.7033 0.1875 0.3557 0.8533 0.9252 >0.9999 | 0.7981 |
| Monocyte count (absolute) [10^3/µl] | 0.5838  (n= 24) | 0.7160 (n= 20) | 0.6470  (n= 20) | 0.6318  (n= 22) | t0 vs. t7 t0 vs. t20  t0 vs. t40  t7 vs. t20 t7 vs. t40 t20 vs. t40 | -0.1323 -0.06325 -0.04807 0.06900 0.08418 0.01518 | -0.2530 to -0.01151 -0.2585 to 0.1320 -0.2074 to 0.1113 -0.07630 to 0.2143 -0.01939 to 0.1878 -0.1539 to 0.1843 | **0.0288** 0.7993 0.8345 0.5413 0.1348 0.9941 | 0.7924 |
| Monocyte count (relative) [%] | 7.663  (n= 24) | 9.545 (n= 20) | 8.460  (n= 20) | 8.286  (n= 22) | t0 vs. t7 t0 vs. t20  t0 vs. t40  t7 vs. t20 t7 vs. t40 t20 vs. t40 | -1.883 -0.7975 -0.6239 1.085 1.259 0.1736 | -3.299 to -0.4658 -2.098 to 0.5033 -1.969 to 0.7209 -0.8611 to 3.031 -0.4563 to 2.974 -1.844 to 2.192 | **0.0070** 0.3393 0.5772 0.4088 0.1972 0.9948 | 0.8332 |
| Erythrocyte distribution width [fl] | 44.65  (n= 24) | 43.94 (n= 20) | 47.39  (n= 20) | 47.64  (n= 22) | t0 vs. t7 t0 vs. t20  t0 vs. t40  t7 vs. t20 t7 vs. t40 t20 vs. t40 | 0.7142 -2.731 -2.982 -3.445 -3.696 -0.2514 | -0.2876 to 1.716 -6.684 to 1.223 -6.020 to 0.05528 -6.313 to -0.5766 -6.306 to -1.086 -1.904 to 1.401 | 0.2212 0.2444 0.0555 **0.0161 0.0044** 0.9726 | 0.3049 |
| Platelet-large cell ratio [%] | 27.73  (n= 24) | 27.20 (n= 20) | 25.02  (n= 20) | 26.84  (n= 22) | t0 vs. t7 t0 vs. t20  t0 vs. t40  t7 vs. t20 t7 vs. t40 t20 vs. t40 | 0.5292 2.709 0.8883 2.180 0.3591 -1.821 | -1.403 to 2.461 0.2340 to 5.184 -1.086 to 2.863 0.1767 to 4.183 -1.565 to 2.283 -3.597 to -0.04520 | 0.8669 **0.0289** 0.6009 **0.0306** 0.9505 **0.0433** | 0.7008 |
| Basophil count (absolute) [10^3/µl] | 0.041  (n= 24) | 0.041  (n= 20) | 0.043  (n= 20) | 0.036  (n= 22) | t0 vs. t7 t0 vs. t20  t0 vs. t40  t7 vs. t20 t7 vs. t40 t20 vs. t40 | 0.0002500 -0.002250 0.004432 -0.002500 0.004182 0.006682 | -0.01291 to 0.01341 -0.01339 to 0.008885 -0.009437 to 0.01830 -0.01448 to 0.009475 -0.005301 to 0.01366 -0.006043 to 0.01941 | >0.9999 0.9403 0.8097 0.9314 0.6030 0.4669 | 0.7040 |
| Basophil count (relative) [%] | 0.545  (n= 24) | 0.565  (n= 20) | 0.5950  (n= 20) | 0.5  (n= 22) | t0 vs. t7 t0 vs. t20  t0 vs. t40  t7 vs. t20 t7 vs. t40 t20 vs. t40 | -0.01917 -0.04917 0.04583 -0.03000 0.06500 0.09500 | -0.2083 to 0.1700 -0.1959 to 0.09758 -0.1539 to 0.2455 -0.2075 to 0.1475 -0.1036 to 0.2336 -0.03116 to 0.2212 | 0.9917 0.7829 0.9179 0.9616 0.6967 0.1819 | 0.7054 |
| Immature granulocyte count [%] | 0.3792  (n= 24) | 0.37  (n= 20) | 0.7650  (n= 20) | 0.504  (n= 22) | t0 vs. t7 t0 vs. t20  t0 vs. t40  t7 vs. t20 t7 vs. t40 t20 vs. t40 | 0.009167 -0.3858 -0.1254 -0.3950 -0.1345 0.2605 | -0.1074 to 0.1257 -1.195 to 0.4230 -0.3288 to 0.07809 -0.7218 to -0.06823 -0.3509 to 0.08177 -0.5374 to 1.058 | 0.9960 0.5493 0.3399 **0.0154** 0.3217 0.7932 | 0.3998 |
| Normoblast count [%] | 0  (n= 24) | 0  (n= 20) | 0.02  (n= 20) | 0  (n= 22) | t0 vs. t7 t0 vs. t20  t0 vs. t40  t7 vs. t20 t7 vs. t40 t20 vs. t40 | 0.000 -0.02000 0.000 -0.02000 0.000 0.02000 | -0.07384 to 0.03384  -0.03404 to -0.005964  -0.03923 to 0.07923 | 0.7261  **0.0049**  0.7734 | 0.3333 |
| Mean platelet volume [fl] | 10.4  (n= 24) | 10.33  (n= 20) | 10.05  (n= 20) | 10.25  (n= 22) | t0 vs. t7 t0 vs. t20  t0 vs. t40  t7 vs. t20 t7 vs. t40 t20 vs. t40 | 0.06583 0.3458 0.1458 0.2800 0.08000 -0.2000 | -0.1779 to 0.3096 0.04446 to 0.6472 -0.09162 to 0.3833 0.02108 to 0.5389 -0.1645 to 0.3245 -0.4457 to 0.04569 | 0.8715 0.0211 0.3427 0.0318 0.7893 0.1350 | 0.6574 |
| Quick [%] | 94.91  (n= 23) | 93.82 (n= 22) | 94.68  (n= 19) | 94.14  (n= 21) | t0 vs. t7 t0 vs. t20  t0 vs. t40  t7 vs. t20 t7 vs. t40 t20 vs. t40 | 1.095 0.2288 0.7702 -0.8660 -0.3247 0.5414 | -2.285 to 4.475 -3.759 to 4.216 -2.401 to 3.942 -3.847 to 2.115 -4.075 to 3.425 -3.626 to 4.709 | 0.8015 0.9984 0.9023 0.8389 0.9947 0.9822 | 0.8822 |
| INR | 1.018  (n= 24) | 1.028  (n= 22) | 1.019  (n= 19) | 1.041  (n= 21) | t0 vs. t7 t0 vs. t20  t0 vs. t40  t7 vs. t20 t7 vs. t40 t20 vs. t40 | -0.009394 -0.0006140 -0.02262 0.008780 -0.01323 -0.02201 | -0.04190 to 0.02312 -0.03919 to 0.03796 -0.05575 to 0.01051 -0.02242 to 0.03998 -0.05413 to 0.02768 -0.06659 to 0.02258 | 0.8511 >0.9999 0.2551 0.8511 0.7978 0.5145 | 0.8893 |
| aPTT [s] | 29.93  (n= 23) | 30.04 (n= 22) | 29.47  (n= 19) | 29.65  (n= 21) | t0 vs. t7 t0 vs. t20  t0 vs. t40  t7 vs. t20 t7 vs. t40 t20 vs. t40 | -0.1059 0.4620 0.2781 0.5679 0.3840 -0.1840 | -1.186 to 0.9737 -1.022 to 1.946 -1.040 to 1.596 -0.2182 to 1.354 -0.7202 to 1.488 -1.162 to 0.7942 | 0.9926 0.8127 0.9329 0.2058 0.7608 0.9494 | 0.4485 |
| Thrombin clotting time [s] | 16.26  (n= 23) | 16.04  (n= 22) | 16.09  (n= 19) | 16.57  (n= 21) | t0 vs. t7 t0 vs. t20  t0 vs. t40  t7 vs. t20 t7 vs. t40 t20 vs. t40 | 0.2202 0.1670 -0.3101 -0.05311 -0.5303 -0.4772 | -0.1943 to 0.6347 -0.3727 to 0.7068 -0.9403 to 0.3200 -0.4855 to 0.3793 -1.120 to 0.05945 -1.158 to 0.2032 | 0.4636 0.8152 0.5270 0.9846 0.0868 0.2289 | 0.7987 |
| Fibrinogen [mg/dl] | 408.3  (n= 23) | 449.6  (n= 22) | 413.3  (n= 18) | 411.7  (n= 20) | t0 vs. t7 t0 vs. t20  t0 vs. t40  t7 vs. t20 t7 vs. t40 t20 vs. t40 | -41.38 -5.017 -3.439 36.36 37.94 1.578 | -91.95 to 9.196 -78.92 to 68.89 -78.86 to 71.98 -29.13 to 101.8 -26.47 to 102.3 -76.37 to 79.52 | 0.1340 0.9973 0.9992 0.4079 0.3667 >0.9999 | 0.7090 |
| D-dimer [ng/ml] | 1418  (n= 24) | 1164  (n= 22) | 1816  (n= 18) | 1123  (n= 21) | t0 vs. t7 t0 vs. t20  t0 vs. t40  t7 vs. t20 t7 vs. t40 t20 vs. t40 | 253.8 -398.2 295.1 -652.0 41.23 693.3 | -307.8 to 815.4 -1103 to 307.0 -564.0 to 1154 -1785 to 480.8 -302.7 to 385.2 -571.9 to 1958 | 0.5974 0.4020 0.7725 0.3778 0.9862 0.4233 | 0.3821 |
| Protein C activity [%] | 107.1  (n= 21) | 100.5  (n= 22) | 104.7  (n= 18) | 109.0  (n= 20) | t0 vs. t7 t0 vs. t20  t0 vs. t40  t7 vs. t20 t7 vs. t40 t20 vs. t40 | 6.597 2.421 -1.857 -4.177 -8.455 -4.278 | -1.595 to 14.79 -9.628 to 14.47 -17.96 to 14.25 -16.84 to 8.489 -22.59 to 5.680 -18.30 to 9.743 | 0.1410 0.9382 0.9871 0.7786 0.3538 0.8154 | 0.7915 |
| Protein S (free antigen) [%] | 101.2  (n= 21) | 102.5  (n= 22) | 99.39  (n= 18) | 95.19  (n= 21) | t0 vs. t7 t0 vs. t20  t0 vs. t40  t7 vs. t20 t7 vs. t40 t20 vs. t40 | -1.262 1.849 6.048 3.111 7.310 4.198 | -10.11 to 7.581 -8.424 to 12.12 -4.725 to 16.82 -6.891 to 13.11 -3.134 to 17.75 -0.5335 to 8.930 | 0.9771 0.9543 0.4069 0.8068 0.2326 0.0914 | 0.1476 |
| Lupus anticoagulant screening [s] | 37.11  (n= 24) | 38.91  (n= 20) | 38.52  (n= 19) | 37.12  (n= 21) | t0 vs. t7 t0 vs. t20  t0 vs. t40  t7 vs. t20 t7 vs. t40 t20 vs. t40 | -1.801 -1.413 -0.01548 0.3880 1.785 1.397 | -4.081 to 0.4794 -5.174 to 2.348 -3.343 to 3.312 -2.288 to 3.065 -0.4170 to 3.988 -1.822 to 4.617 | 0.1555 0.7164 >0.9999 0.9751 0.1373 0.6150 | 0.6775 |
| Lupus anticoagulant PTT [s] | 37.76  (n= 23) | 37.77  (n= 22) | 37.05  (n= 19) | 36.93  (n= 21) | t0 vs. t7 t0 vs. t20  t0 vs. t40  t7 vs. t20 t7 vs. t40 t20 vs. t40 | -0.007312 0.7082 0.8323 0.7156 0.8396 0.1241 | -1.851 to 1.837 -1.720 to 3.137 -2.040 to 3.704 -0.4832 to 1.914 -0.7612 to 2.440 -1.175 to 1.423 | >0.9999 0.8400 0.8467 0.3519 0.4678 0.9927 | 0.2416 |
| Lupus anticoagulant ICA (Index of circulating anticoagulant) [s] | 61.07  (n= 24) | 62.81  (n= 22) | 64.98  (n= 19) | 60.93  (n= 21) | t0 vs. t7 t0 vs. t20  t0 vs. t40  t7 vs. t20 t7 vs. t40 t20 vs. t40 | -1.747 -3.918 0.1333 -2.171 1.880 4.051 | -6.576 to 3.082 -11.02 to 3.189 -8.195 to 8.462 -9.861 to 5.520 -6.297 to 10.06 -0.9246 to 9.026 | 0.7463 0.4258 >0.9999 0.8500 0.9142 0.1338 | 0.3557 |
| Lupus anticoagulant ICA (Index of circulating anticoagulant) 1+1 [s] | 56.18  (n= 24) | 57.15  (n= 22) | 59.18  (n= 19) | 56.82  (n= 21) | t0 vs. t7 t0 vs. t20  t0 vs. t40  t7 vs. t20 t7 vs. t40 t20 vs. t40 | -0.9667 -2.996 -0.6357 -2.029 0.3310 2.360 | -4.160 to 2.227 -6.782 to 0.7911 -5.583 to 4.312 -7.495 to 3.437 -5.890 to 6.552 -0.8792 to 5.599 | 0.8330 0.1513 0.9836 0.7165 0.9987 0.2022 | 0.1843 |
| Lupus anticoagulant ICA NP [s] | 51.55  (n= 24) | 51.63  (n= 22) | 51.88  (n= 19) | 52.04  (n= 21) | t0 vs. t7 t0 vs. t20  t0 vs. t40  t7 vs. t20 t7 vs. t40 t20 vs. t40 | -0.08598 -0.3384 -0.4970 -0.2524 -0.4110 -0.1586 | -1.546 to 1.374 -2.182 to 1.505 -2.293 to 1.299 -1.785 to 1.280 -2.077 to 1.255 -1.730 to 1.413 | 0.9984 0.9535 0.8649 0.9643 0.8968 0.9915 | 0.9316 |
| Cardiolipin-IgG-antibody [GPL U/ml] | 8.527  (n= 22) | 9.943  (n= 21) | 5.917  (n= 18) | 11.29  (n= 21) | t0 vs. t7 t0 vs. t20  t0 vs. t40  t7 vs. t20 t7 vs. t40 t20 vs. t40 | -1.416 2.611 -2.758 4.026 -1.343 -5.369 | -5.924 to 3.092 -1.706 to 6.928 -10.48 to 4.963 -3.888 to 11.94 -5.439 to 2.753 -16.85 to 6.116 | 0.8113 0.3412 0.7487 0.4805 0.7912 0.5489 | 0.3453 |
| Cardiolipin-IgM-antibody [MPL U/ml] | 8.609  (n= 22) | 8.138  (n= 21) | 6.000  (n= 18) | 11.85  (n= 21) | t0 vs. t7 t0 vs. t20  t0 vs. t40  t7 vs. t20 t7 vs. t40 t20 vs. t40 | 0.4710 2.609 -3.243 2.138 -3.714 -5.852 | -5.453 to 6.395 -3.228 to 8.446 -9.417 to 2.930 -5.306 to 9.582 -11.29 to 3.862 -18.67 to 6.965 | 0.9958 0.5886 0.4698 0.8405 0.5238 0.5674 | 0.02496 |
| β2-glycoprotein-IgG antibody [U/ml] | 3.127  (n= 22) | 2.823  (n= 21) | 1.967  (n= 18) | 2.576  (n= 21) | t0 vs. t7 t0 vs. t20  t0 vs. t40  t7 vs. t20 t7 vs. t40 t20 vs. t40 | 0.304 1.16 0.551 0.856 0.247 -0.609 | -1.337 to 2.023 -1.667 to 3.749 -2.009 to 2.998 -0.9897 to 2.386 -1.034 to 1.338 -1.904 to 0.8123 | 0.9396 0.7021 0.9453 0.6495 0.9837 0.6692 | 0.4297 |
| β2-glycoprotein-IgM antibody [U/ml] | 5.209  (n= 22) | 6.340  (n= 20) | 4.453  (n= 17) | 6.005  (n= 21) | t0 vs. t7 t0 vs. t20  t0 vs. t40  t7 vs. t20 t7 vs. t40 t20 vs. t40 | -1.131 0.7561 -0.7957 1.887 0.3352 -1.552 | -4.949 to 2.688 -4.262 to 5.775 -4.183 to 2.592 -3.983 to 7.757 -1.403 to 2.074 -5.628 to 2.524 | 0.8339 0.9716 0.9106 0.7825 0.9458 0.6915 | 0.3152 |
| Platelet aggregation ADP [%] | 67.60  (n= 21) | 65.65  (n= 20) | 68.53  (n= 17) | 63.68  (n= 19) | t0 vs. t7 t0 vs. t20  t0 vs. t40  t7 vs. t20 t7 vs. t40 t20 vs. t40 | 1.950 -0.9294 3.916 -2.879 1.966 4.845 | -11.01 to 14.91 -12.43 to 10.57 -9.194 to 17.03 -15.54 to 9.786 -11.56 to 15.49 -4.535 to 14.23 | 0.9724 0.9952 0.8304 0.9075 0.9744 0.4680 | 0.8187 |
| Platelet Aggrgation collagen [%] | 64.24  (n= 21) | 61.05  (n= 20) | 63.18  (n= 17) | 61.53  (n= 19) | t0 vs. t7 t0 vs. t20  t0 vs. t40  t7 vs. t20 t7 vs. t40 t20 vs. t40 | 3.188 1.062 2.712 -2.126 -0.4763 1.650 | -7.479 to 13.86 -5.572 to 7.696 -2.245 to 7.669 -15.07 to 10.82 -13.58 to 12.63 -4.646 to 7.946 | 0.8274 0.9655 0.4287 0.9617 0.9996 0.8730 | 0.5345 |
| Platelet aggregation Ristocetin 1,2 [%] | 66.30  (n= 20) | 64.65  (n= 20) | 66.80  (n= 15) | 62.72  (n= 18) | t0 vs. t7 t0 vs. t20  t0 vs. t40  t7 vs. t20 t7 vs. t40 t20 vs. t40 | 1.650 -0.5000 3.578 -2.150 1.928 4.078 | -10.40 to 13.70 -6.714 to 5.714 -3.739 to 10.89 -16.21 to 11.91 -12.83 to 16.69 -3.773 to 11.93 | 0.9789 0.9949 0.5178 0.9675 0.9811 0.4518 | 0.7065 |
| Platelet aggreagtion Ristocetin 0,6 [%] | 2.333  (n= 21) | 3.2  (n= 20) | 3.063  (n= 16) | 3.368  (n= 19) | t0 vs. t7 t0 vs. t20  t0 vs. t40  t7 vs. t20 t7 vs. t40 t20 vs. t40 | -0.8667 -0.7292 -1.035 0.1375 -0.1684 -0.3059 | -2.450 to 0.7164 -1.984 to 0.5256 -3.959 to 1.889 -2.611 to 2.886 -3.285 to 2.948 -2.602 to 1.990 | 0.4241 0.3597 0.7479 0.9988 0.9986 0.9795 | 0.5859 |
| Platelet aggregometry (PFA)  Collagen/epinephrin [s] | 153.4  (n= 24) | 137.8  (n= 21) | 143  (n= 18) | 131.8  (n= 19) | t0 vs. t7 t0 vs. t20  t0 vs. t40  t7 vs. t20 t7 vs. t40 t20 vs. t40 | 15.57 10.38 21.59 -5.190 6.020 11.21 | -7.562 to 38.69 -13.10 to 33.85 -0.4990 to 43.67 -22.57 to 12.19 -19.13 to 31.17 -12.00 to 34.42 | 0.2662 0.6014 0.0567 0.8246 0.9014 0.5229 | 0.7650 |
| Platelet aggregometry (PFA)  Collagen/ADP [s] | 98.08  (n= 24) | 94.81  (n= 21) | 92.67  (n= 18) | 93.84  (n= 19) | t0 vs. t7 t0 vs. t20  t0 vs. t40  t7 vs. t20 t7 vs. t40 t20 vs. t40 | 3.274 5.417 4.241 2.143 0.9674 -1.175 | -4.272 to 10.82 -5.586 to 16.42 -7.238 to 15.72 -11.77 to 16.05 -11.13 to 13.06 -16.64 to 14.29 | 0.6252 0.5165 0.7263 0.9698 0.9956 0.9961 | 0.6570 |
| EXTEM Clotting Time (CT) [s] | 65.0  (n= 24) | 69.35  (n= 20) | 65.68  (n= 19) | 64.67  (n= 21) | t0 vs. t7 t0 vs. t20  t0 vs. t40  t7 vs. t20 t7 vs. t40 t20 vs. t40 | -4.350 -0.6842 0.3333 3.666 4.683 1.018 | -9.648 to 0.9481 -5.662 to 4.293 -7.388 to 8.054 -2.354 to 9.685 -3.284 to 12.65 -6.487 to 8.522 | 0.1312 0.9795 0.9993 0.3355 0.3683 0.9799 | 0.6946 |
| EXTEM clot formation time (CFT) [s] | 72.4  (n= 24) | 75.15  (n= 20) | 68.74  (n= 19) | 70.71  (n= 22) | t0 vs. t7 t0 vs. t20  t0 vs. t40  t7 vs. t20 t7 vs. t40 t20 vs. t40 | -2.692 3.721 1.744 6.413 4.436 -1.977 | -11.60 to 6.220 -11.27 to 18.71 -10.43 to 13.91 -4.093 to 16.92 -6.574 to 15.45 -19.26 to 15.30 | 0.8303 0.8951 0.9776 0.3335 0.6677 0.9877 | 0.6729 |
| EXTEM alpha [°] | 76.17  (n= 24) | 75.55  (n= 20) | 76.89  (n= 19) | 76.05  (n= 22) | t0 vs. t7 t0 vs. t20  t0 vs. t40  t7 vs. t20 t7 vs. t40 t20 vs. t40 | 0.6167 -0.7281 0.1190 -1.345 -0.4976 0.8471 | -1.424 to 2.657 -3.882 to 2.426 -2.217 to 2.455 -3.352 to 0.6620 -2.567 to 1.571 -2.605 to 4.300 | 0.8301 0.9133 0.9989 0.2600 0.9019 0.8966 | 0.7019 |
| EXTEM A10 [mm] | 60.29  (n= 24) | 61.05  (n= 20) | 61.63  (n= 19) | 61.62  (n= 21) | t0 vs. t7 t0 vs. t20  t0 vs. t40  t7 vs. t20 t7 vs. t40 t20 vs. t40 | -0.7583 -1.340 -1.327 -0.5816 -0.5690 0.01253 | -3.718 to 2.201 -5.628 to 2.948 -5.258 to 2.604 -3.656 to 2.492 -3.948 to 2.810 -4.492 to 4.517 | 0.8877 0.8135 0.7812 0.9476 0.9628 >0.9999 | 0.7985 |
| EXTEM A20 [mm] | 65.88  (n= 24) | 66.80  (n= 20) | 66.95  (n= 19) | 66.95  (n= 21) | t0 vs. t7 t0 vs. t20  t0 vs. t40  t7 vs. t20 t7 vs. t40 t20 vs. t40 | -0.9250 -1.072 -1.077 -0.1474 -0.1524 -0.005013 | -3.463 to 1.613 -5.051 to 2.906 -4.557 to 2.402 -2.825 to 2.531 -3.111 to 2.806 -3.992 to 3.982 | 0.7372 0.8704 0.8218 0.9985 0.9988 >0.9999 | 0.7750 |
| EXTEM maximum clot firmness (MCF) [mm] | 66.54  (n= 24) | 67.35  (n= 20) | 67.58  (n= 19) | 67.67  (n= 21) | t0 vs. t7 t0 vs. t20  t0 vs. t40  t7 vs. t20 t7 vs. t40 t20 vs. t40 | -0.8083 -1.037 -1.125 -0.2289 -0.3167 -0.08772 | -3.189 to 1.573 -4.643 to 2.568 -4.392 to 2.142 -2.641 to 2.183 -3.125 to 2.492 -3.779 to 3.603 | 0.7761 0.8475 0.7711 0.9927 0.9882 0.9999 | 0.7596 |
| EXTEM maximum lysis (ML) [°] | 6.792  (n= 24) | 7.850  (n= 20) | 8.053  (n= 19) | 7.190  (n= 21) | t0 vs. t7 t0 vs. t20  t0 vs. t40  t7 vs. t20 t7 vs. t40 t20 vs. t40 | -1.058 -1.261 -0.3988 -0.2026 0.6595 0.8622 | -3.294 to 1.177 -3.775 to 1.253 -2.789 to 1.991 -2.321 to 1.916 -0.9558 to 2.275 -2.064 to 3.788 | 0.5553 0.5052 0.9654 0.9925 0.6586 0.8359 | 0.8567 |
| INTEM Clotting Time (CT) [s] | 165.6  (n= 24) | 172.0  (n= 20) | 169.4  (n= 19) | 173.6  (n= 21) | t0 vs. t7 t0 vs. t20  t0 vs. t40  t7 vs. t20 t7 vs. t40 t20 vs. t40 | -6.417 -3.838 -8.036 2.579 -1.619 -4.198 | -19.89 to 7.061 -16.45 to 8.772 -19.10 to 3.031 -6.347 to 11.50 -19.91 to 16.67 -21.29 to 12.89 | 0.5508 0.8250 0.2097 0.8411 0.9942 0.8963 | 0.6051 |
| INTEM alpha [°] | 79.58  (n= 24) | 78.40  (n= 20) | 79.11  (n= 19) | 79.71  (n= 21) | t0 vs. t7 t0 vs. t20  t0 vs. t40  t7 vs. t20 t7 vs. t40 t20 vs. t40 | 1.183 0.4781 -0.1310 -0.7053 -1.314 -0.6090 | -0.1959 to 2.563 -1.766 to 2.722 -1.548 to 1.286 -1.808 to 0.3978 -2.721 to 0.09215 -2.582 to 1.364 | 0.1085 0.9301 0.9937 0.2963 0.0716 0.8163 | 0.4831 |
| INTEM A10 [mm] | 60.25  (n= 24) | 60.9  (n= 20) | 61.42  (n= 19) | 61.67  (n= 21) | t0 vs. t7 t0 vs. t20  t0 vs. t40  t7 vs. t20 t7 vs. t40 t20 vs. t40 | -0.6500 -1.171 -1.417 -0.5211 -0.7667 -0.2456 | -2.916 to 1.616 -4.876 to 2.534 -4.330 to 1.496 -3.261 to 2.219 -3.125 to 1.592 -3.344 to 2.853 | 0.8505 0.8083 0.5368 0.9468 0.7925 0.9958 | 0.7568 |
| INTEM A20 [mm] | 64.42  (n= 24) | 65.1  (n= 20) | 65.37  (n= 19) | 65.5  (n= 21) | t0 vs. t7 t0 vs. t20  t0 vs. t40  t7 vs. t20 t7 vs. t40 t20 vs. t40 | -0.6833 -0.9518 -1.083 -0.2684 -0.4000 -0.1316 | -2.701 to 1.335 -4.222 to 2.318 -3.774 to 1.608 -2.855 to 2.319 -2.611 to 1.811 -2.913 to 2.650 | 0.7775 0.8431 0.6749 0.9905 0.9545 0.9991 | 0.7923 |
| INTEM maximum clot firmness (MCF) [mm] | 64.58  (n= 24) | 65.1  (n= 20) | 65.47  (n= 19) | 65.81  (n= 21) | t0 vs. t7 t0 vs. t20  t0 vs. t40  t7 vs. t20 t7 vs. t40 t20 vs. t40 | -0.5167 -0.8904 -1.226 -0.3737 -0.7095 -0.3358 | -2.563 to 1.530 -4.088 to 2.308 -3.891 to 1.438 -2.887 to 2.140 -2.938 to 1.519 -3.071 to 2.400 | 0.8919 0.8594 0.5807 0.9733 0.8024 0.9849 | 0.7961 |
| INTEM maximum lysis (ML) [°] | 7.958  (n= 24) | 9.5  (n= 20) | 14.0  (n= 19) | 8.238  (n= 21) | t0 vs. t7 t0 vs. t20  t0 vs. t40  t7 vs. t20 t7 vs. t40 t20 vs. t40 | -1.542 -6.042 -0.2798 -4.500 1.262 5.762 | -3.856 to 0.7728 -20.30 to 8.218 -2.733 to 2.173 -19.74 to 10.74 -0.8180 to 3.342 -8.876 to 20.40 | 0.2722 0.6362 0.9884 0.8324 0.3420 0.6832 | 0.3613 |
| FIBTEM Clotting Time (CT) [s] | 97.29  (n= 24) | 64.9  (n= 20) | 60.26  (n= 19) | 59.43  (n= 21) | t0 vs. t7 t0 vs. t20  t0 vs. t40  t7 vs. t20 t7 vs. t40 t20 vs. t40 | 33.29 37.03 37.86 3.737 4.571 0.8346 | -87.42 to 154.0 -89.61 to 163.7 -78.89 to 154.6 -2.129 to 9.603 -3.179 to 12.32 -6.028 to 7.697 | 0.8645 0.8412 0.8010 0.2993 0.3654 0.9853 | 0.3361 |
| FIBTEM alpha [°] | 76.0  (n= 23) | 75.6  (n= 20) | 77.58  (n= 19) | 75.57  (n= 21) | t0 vs. t7 t0 vs. t20  t0 vs. t40  t7 vs. t20 t7 vs. t40 t20 vs. t40 | 0.4000 -1.579 0.4286 -1.979 0.02857 2.008 | -2.436 to 3.236 -4.680 to 1.522 -3.143 to 4.000 -5.150 to 1.192 -2.155 to 2.212 -2.393 to 6.408 | 0.9779 0.4889 0.9864 0.3156 >0.9999 0.5772 | 0.7632 |
| FIBTEM A10 [mm] | 21.75  (n= 24) | 24.7  (n= 20) | 24.79  (n= 19) | 23.0  (n= 21) | t0 vs. t7 t0 vs. t20  t0 vs. t40  t7 vs. t20 t7 vs. t40 t20 vs. t40 | -2.950 -3.039 -1.250 -0.08947 1.700 1.789 | -6.749 to 0.8486 -8.067 to 1.988 -6.082 to 3.582 -3.886 to 3.707 -2.572 to 5.972 -2.377 to 5.956 | 0.1637 0.3482 0.8863 0.9999 0.6761 0.6227 | 0.8643 |
| FIBTEM A20 [mm] | 22.54  (n= 24) | 25.95  (n= 20) | 26.3  (n= 20) | 23.95  (n= 21) | t0 vs. t7 t0 vs. t20  t0 vs. t40  t7 vs. t20 t7 vs. t40 t20 vs. t40 | -3.408 -3.758 -1.411 -0.3500 1.998 2.348 | -7.719 to 0.9026 -8.859 to 1.342 -6.522 to 3.701 -4.355 to 3.655 -2.404 to 6.399 -1.738 to 6.433 | 0.1528 0.1963 0.8643 0.9943 0.5812 0.3874 | 0.8876 |
| FIBTEM maximum clot firmness (MCF) [mm] | 22.79  (n= 24) | 26.0  (n= 20) | 26.16  (n= 19) | 23.9  (n= 21) | t0 vs. t7 t0 vs. t20  t0 vs. t40  t7 vs. t20 t7 vs. t40 t20 vs. t40 | -3.208 -3.366 -1.113 -0.1579 2.095 2.253 | -7.340 to 0.9237 -8.319 to 1.586 -5.973 to 3.747 -4.122 to 3.806 -2.326 to 6.517 -2.034 to 6.541 | 0.1638 0.2544 0.9174 0.9994 0.5473 0.4625 | 0.8987 |
| FIBTEM maximum lysis (ML) [°] | 1.75  (n= 24) | 2.4  (n= 20) | 8.105  (n= 19) | 2.0  (n= 21) | t0 vs. t7 t0 vs. t20  t0 vs. t40  t7 vs. t20 t7 vs. t40 t20 vs. t40 | -0.6500 -6.355 -0.2500 -5.705 0.4000 6.105 | -2.267 to 0.9673 -20.07 to 7.363 -1.971 to 1.471 -20.74 to 9.328 -1.779 to 2.579 -8.583 to 20.79 | 0.6761 0.5690 0.9767 0.7029 0.9527 0.6461 | 0.3487 |
| APTEM Clotting Time (CT) [s] | 60.92  (n= 24) | 67.1  (n= 20) | 63.11  (n= 19) | 60.1  (n= 21) | t0 vs. t7 t0 vs. t20  t0 vs. t40  t7 vs. t20 t7 vs. t40 t20 vs. t40 | -6.183 -2.189 0.8214 3.995 7.005 3.010 | -11.74 to -0.6285 -7.147 to 2.770 -4.973 to 6.616 -1.526 to 9.516 0.3273 to 13.68 -2.731 to 8.751 | **0.0259** 0.6061 0.9782 0.2048 **0.0380** 0.4645 | 0.8581 |
| APTEM clot formation time (CFT) [s] | 69.38  (n= 24) | 68.75  (n= 20) | 66.53  (n= 19) | 66.81  (n= 21) | t0 vs. t7 t0 vs. t20  t0 vs. t40  t7 vs. t20 t7 vs. t40 t20 vs. t40 | 0.6250 2.849 2.565 2.224 1.940 -0.2832 | -13.59 to 14.84 -12.97 to 18.66 -6.334 to 11.47 -10.03 to 14.47 -8.116 to 12.00 -10.62 to 10.06 | 0.9993 0.9559 0.8504 0.9532 0.9457 0.9998 | 0.5815 |
| APTEM alpha [°] | 76.58  (n= 24) | 76.05  (n= 20) | 77.0  (n= 19) | 76.81  (n= 21) | t0 vs. t7 t0 vs. t20  t0 vs. t40  t7 vs. t20 t7 vs. t40 t20 vs. t40 | 0.5333 -0.4167 -0.2262 -0.9500 -0.7595 0.1905 | -1.786 to 2.853 -4.174 to 3.341 -2.170 to 1.718 -3.177 to 1.277 -2.425 to 0.9065 -1.891 to 2.272 | 0.9154 0.9890 0.9877 0.6234 0.5777 0.9936 | 0.5272 |
| APTEM A10 [mm] | 60.38  (n= 24) | 60.7  (n= 20) | 61.58  (n= 19) | 61.43  (n= 21) | t0 vs. t7 t0 vs. t20  t0 vs. t40  t7 vs. t20 t7 vs. t40 t20 vs. t40 | -0.3250 -1.204 -1.054 -0.8789 -0.7286 0.1504 | -3.022 to 2.372 -5.222 to 2.814 -4.198 to 2.091 -3.768 to 2.010 -2.997 to 1.540 -2.850 to 3.151 | 0.9862 0.8315 0.7852 0.8199 0.7984 0.9989 | 0.7720 |
| APTEM A20 [mm] | 65.58  (n= 24) | 66.1  (n= 20) | 66.68  (n= 19) | 66.35  (n= 20) | t0 vs. t7 t0 vs. t20  t0 vs. t40  t7 vs. t20 t7 vs. t40 t20 vs. t40 | -0.5167 -1.101 -0.7667 -0.5842 -0.2500 0.3342 | -2.601 to 1.568 -4.739 to 2.537 -3.526 to 1.993 -3.206 to 2.037 -2.255 to 1.755 -2.412 to 3.080 | 0.8970 0.8274 0.8620 0.9183 0.9842 0.9850 | 0.6989 |
| APTEM maximum clot firmness (MCF) [mm] | 66.25  (n= 24) | 66.65  (n= 20) | 67.26  (n= 19) | 66.9  (n= 21) | t0 vs. t7 t0 vs. t20  t0 vs. t40  t7 vs. t20 t7 vs. t40 t20 vs. t40 | -0.4000 -1.013 -0.6548 -0.6132 -0.2548 0.3584 | -2.495 to 1.695 -4.357 to 2.331 -3.410 to 2.101 -2.856 to 1.630 -2.090 to 1.580 -2.089 to 2.806 | 0.9489 0.8268 0.9090 0.8615 0.9785 0.9749 | 0.6947 |
| APTEM maximum lysis (ML) [°] | 7.0  (n= 24) | 8.850  (n= 20) | 12.89  (n= 19) | 7.762  (n= 21) | t0 vs. t7 t0 vs. t20  t0 vs. t40  t7 vs. t20 t7 vs. t40 t20 vs. t40 | -1.850 -5.895 -0.7619 -4.045 1.088 5.133 | -4.338 to 0.6380 -20.13 to 8.340 -2.630 to 1.106 -19.53 to 11.44 -0.3589 to 2.535 -9.716 to 19.98 | 0.1918 0.6524 0.6690 0.8764 0.1812 0.7610 | 0.3548 |
| P-Selectin [pg/ml] | 3565.123  (n= 8) | 3015.28  (n= 8) | 4530.59  (n= 6) | 3639.1  (n= 8) | t0 vs. t7 t0 vs. t20  t0 vs. t40  t7 vs. t20 t7 vs. t40 t20 vs. t40 | 549.843  -965.467  -73.977  -1515.31  -623.82  891.49 | -911.4 to 2011  -3125 to 1501  -773.4 to 625.0  -4514 to 1495  -2305 to 1057  -1177 to 2256 | 0.6207  0.6031  0.9839  0.2510  0.6299  0.6738 | 0.4320 |
| tPA [pg/ml] | 4893.79  (n= 8) | 5210.22  (n= 8) | 5600.03  (n= 6) | 5802.19  (n= 8) | t0 vs. t7 t0 vs. t20  t0 vs. t40  t7 vs. t20 t7 vs. t40 t20 vs. t40 | -316.42  -706.24  -908.4  -389.81  -591.97  -202.16 | -1583 to 950.4  -2429 to 673.8  -2803 to 986.1  - 1703 to 641.9  -2288 to 1104  -1870 to 499.2 | 0.8404  0.2735  0.4424  0.4240  0.6704  0.2598 | 0.5684 |
| sCD40L [pg/ml] | 1186.77  (n= 8) | 670.91  (n= 8) | 682.69  (n= 6) | 629.65  (n= 8) | t0 vs. t7 t0 vs. t20  t0 vs. t40  t7 vs. t20 t7 vs. t40 t20 vs. t40 | 515.86  504.04  557.12  -11.78  41.26  53.04 | -1033 to 2064  -1556 to 2903  -1130 to 2244  -535.5 to 386.9  -325.2 to 407.8  -178.2 to 345.8 | 0.6993  0.6974  0.7045  0.9296  0.9809  0.6631 | 0.3601 |
| PAI-1 [pg/ml] | 46903.74  (n= 8) | 55264.69  (n= 8) | 53566.11  (n= 6) | 57080.34  (n= 8) | t0 vs. t7 t0 vs. t20  t0 vs. t40  t7 vs. t20 t7 vs. t40 t20 vs. t40 | -8360.96  -6662.37  -10176.6  1698.58  -1815.65  -3514.23 | -20922 to 4200  -28342 to 7975  -28036 to 7683  -8168 to 2441  -17615 to 13983  -16522 to 4893 | 0.2118  0.2789  0.3138  0.3032  0.9798  0.2992 | 0.5480 |
| Factor IX [pg/ml] | 227236  (n= 8) | 236771  (n= 8) | 205559  (n= 6) | 241406  (n= 8) | t0 vs. t7 t0 vs. t20  t0 vs. t40  t7 vs. t20 t7 vs. t40 t20 vs. t40 | -9535  21677  -14170  31212  -4635  -35847 | -89191 to 70121  -75869 to 115120  -110246 to 81906  -72713 to 99988  -82049 to 72778  -118565 to 46988 | 0,9773  0,8698  0,9593  0,9331  0,9970  0,4566 | 0.7331 |
| CXCL8 (IL-8) [pg/ml] | 68.44  (n= 8) | 90.76  (n= 8) | 95.09  (n= 6) | 78.21  (n= 8) | t0 vs. t7 t0 vs. t20  t0 vs. t40  t7 vs. t20 t7 vs. t40 t20 vs. t40 | -22.32  -26.65  -9,77  -4.33  12.55  16.88 | -80,81 to 36,17  -69,65 to 19,62  -69,78 to 50,24  -66,25 to 39,84  -15,45 to 40,55  -30,21 to 67,60 | 0,6111  0,2792  0,9466  0,7973  0,4936  0,5443 | 0.4703 |
|  |  |  |  |  |  |  |  |  |  |
| Blood group | A (n= 4)  B (n= 1)  0 (n= 2) | CC D ee (n= 3)  Cc D ee (n= 2)  Cc D Ee (n= 1)  Cc dd ee (n= 1) | Kell neg (n= 7) | Irregular Antibodies (n=0) |  |  |  |  |  |

^*^Significant *p*-values are written in bold font

**Supplementary Table 3: Measured mean values under BRAF/MEK inhibitor therapy**

|  | **t0 (n)** | **t7 (n)** | **t20 (n)** | **t40 (n)** | **Comparison** | **Mean Diff.** | **95% CI of diff.** | **Adj. *p*-values** | **Geisser-Greenhouse’s epsilon** |
| --- | --- | --- | --- | --- | --- | --- | --- | --- | --- |
| Factor VIII:C [%} | 168.6  (n= 7) | 167.5  (n= 6) | 215.8  (n= 6) | 192.8  (n= 5) | t0 vs. t7 t0 vs. t20  t0 vs. t40  t7 vs. t20 t7 vs. t40 t20 vs. t40 | 1.071  -47.26  -24.23  -48.33  -25.30  23.03 | -62.21 to 64.36  -126.7 to 32.18  -124.9 to 76.44  -98.86 to 2.194  -91.78 to 41.18  -64.60 to 110.7 | >0.9999  0.2433  0.7685  0.0587  0.4912  0.7237 | 0.7424 |
| vWF antigen [%] | 129.4  (n= 7) | 140.2  (n= 6) | 200.7  (n= 6) | 172.2  (n= 5) | t0 vs. t7 t0 vs. t20  t0 vs. t40  t7 vs. t20 t7 vs. t40 t20 vs. t40 | -10.74  -71.24  -42.77  -60.50  -32.03  28.47 | -33.20 to 11.72  -178.7 to 36.26  -139.5 to 53.98  -160.0 to 38.97  -135.5 to 71.44  -126.4 to 183.4 | 0.3851  0.1851  0.3898  0.2306  0.6279  0.8734 | 0.5731 |
| vWF activity [%] | 139.7  (n= 7) | 156.3  (n= 6) | 178.8  (n= 6) | 158.4  (n= 5) | t0 vs. t7 t0 vs. t20  t0 vs. t40  t7 vs. t20 t7 vs. t40 t20 vs. t40 | -16.62  -39.12  -18.69  -22.50  -2.067  20.43 | -42.00 to 8.763  -82.27 to 4.036  -53.63 to 16.26  -59.96 to 14.96  -32.01 to 27.87  -28.89 to 69.75 | 0.1912  0.0708  0.2715  0.2377  0.9911  0.4333 | 0.6491 |
| Antithrombin activity [%] | 104.7  (n= 7) | 102.6  (n= 5) | 107.5  (n= 6) | 105.2  (n= 5) | t0 vs. t7 t0 vs. t20  t0 vs. t40  t7 vs. t20 t7 vs. t40 t20 vs. t40 | 2.114  -2.786  -0.4857  -4.900  -2.600  2.300 | -14.91 to 19.14  -13.29 to 7.722  -12.14 to 11.17  -15.66 to 5.861  -16.79 to 11.59  -7.438 to 12.04 | 0.9536  0.7677  0.9980  0.3703  0.8150  0.7774 | 0.1920 |
| Protein S activity [%] | 112.4  (n= 7) | 106.8  (n= 6) | 87.67  (n= 6) | 108.4  (n= 5) | t0 vs. t7 t0 vs. t20  t0 vs. t40  t7 vs. t20 t7 vs. t40 t20 vs. t40 | 5.595  24.76  4.029  19.17  -1.567  -20.73 | -38.45 to 49.65  -12.15 to 61.67  -48.55 to 56.61  3.940 to 34.39  -28.83 to 25.70  -51.98 to 10.52 | 0.9628  0.1791  0.9880  **0.0206**  0.9948  0.1642 | 0.4505 |
| INTEM Clot formation Time [sec.] | 59.57  (n= 7) | 53.0  (n= 6) | 60.0  (n= 6) | 60.60  (n= 5) | t0 vs. t7 t0 vs. t20  t0 vs. t40  t7 vs. t20 t7 vs. t40 t20 vs. t40 | 6.571  -0.4286  -1.029  -7.000  -7.600  -0.6000 | -9.072 to 22.21  -21.05 to 20.19  -28.20 to 26.14  -35.93 to 21.93  -41.33 to 26.13  -45.89 to 44.69 | 0.4773  0.9998  0.9985  0.8096  0.7986  >0.9999 | 0.7036 |
| FIBTEM Clot formation time [sec.] | 243.0  (n= 4) | 132.3  (n= 4) | 190.8  (n= 4) | 244.7  (n= 3) | t0 vs. t7 t0 vs. t20  t0 vs. t40  t7 vs. t20 t7 vs. t40 t20 vs. t40 | 110.8  52.25  -1.667  -58.50  -112.4  -53.92 | -340.7 to 562.2  -415.6 to 520.1  -5966 to 5963  -423.7 to 306.7  -3192 to 2967  -1215 to 1108 | 0.6744  0.8623  >0.9999  0.7209  0.8582  0.9855 | 0.2665 |
|  |  |  |  |  |  |  |  |  |  |
| Leukocyte count [10^3/µl] | 6.736  (n= 7) | 6.110  (n= 6) | 5.152  (n= 6) | 5.81  (n= 5) | t0 vs. t7 t0 vs. t20  t0 vs. t40  t7 vs. t20 t7 vs. t40 t20 vs. t40 | 0.6257 1.584 0.9257 0.9583 0.3000 -0.6583 | -0.2243 to 1.476 0.3259 to 2.842 -0.4818 to 2.333 -0.1973 to 2.114 -0.9882 to 1.588 -2.516 to 1.199 | 0.1378 **0.0205** 0.1679 0.0953 0.7839 0.5395 | 0.5871 |
| Erythrocyte count [10^6/µl] | 4.849  (n= 7) | 4.828  (n= 6) | 4.815  (n= 6) | 4.740  (n= 5) | t0 vs. t7 t0 vs. t20  t0 vs. t40  t7 vs. t20 t7 vs. t40 t20 vs. t40 | 0.02024 0.03357 0.1086 0.01333 0.08833 0.07500 | -0.3969 to 0.4373 -0.4085 to 0.4756 -0.1499 to 0.3670 -0.4878 to 0.5145 -0.4648 to 0.6415 -0.3332 to 0.4832 | 0.9977 0.9914 0.4239 0.9996 0.9104 0.8735 | 0.4026 |
| Hemoglobin [g/dl] | 14.27  (n= 7) | 14.23  (n= 6) | 14.2  (n= 6) | 14.02  (n= 5) | t0 vs. t7 t0 vs. t20  t0 vs. t40  t7 vs. t20 t7 vs. t40 t20 vs. t40 | 0.03810 0.07143 0.2514 0.03333 0.2133 0.1800 | -1.106 to 1.182 -1.159 to 1.302 -0.5529 to 1.056 -1.411 to 1.477 -1.893 to 2.319 -1.386 to 1.746 | 0.9992 0.9961 0.6218 0.9997 0.9734 0.9624 | 0.4257 |
| Hematocrit [%] | 42.44  (n= 7) | 42.48  (n= 6) | 42.3  (n= 6) | 42.46  (n= 5) | t0 vs. t7 t0 vs. t20  t0 vs. t40  t7 vs. t20 t7 vs. t40 t20 vs. t40 | -0.04048 0.1429 -0.01714 0.1833 0.02333 -0.1600 | -3.434 to 3.353 -3.818 to 4.104 -3.719 to 3.685 -3.851 to 4.218 -5.479 to 5.526 -4.496 to 4.176 | >0.9999 0.9990 >0.9999 0.9981 >0.9999 0.9986 | 0.5541 |
| Platelet count [10^3/µl] | 239.7  (n= 7) | 267.7  (n= 6) | 220.0  (n= 6) | 256.8  (n= 5) | t0 vs. t7 t0 vs. t20  t0 vs. t40  t7 vs. t20 t7 vs. t40 t20 vs. t40 | -27.95 19.71 -17.09 47.67 10.87 -36.80 | -108.6 to 52.72 -45.94 to 85.37 -166.2 to 132.0 -25.13 to 120.5 -52.05 to 73.78 -189.5 to 115.9 | 0.6115 0.7008 0.9627 0.1911 0.8911 0.7679 | 0.3705 |
| MCH [pg] | 29.43  (n= 7) | 29.5  (n= 6) | 29.48  (n= 6) | 29.58  (n= 5) | t0 vs. t7 t0 vs. t20  t0 vs. t40  t7 vs. t20 t7 vs. t40 t20 vs. t40 | -0.07143 -0.05476 -0.1514 0.01667 -0.08000 -0.09667 | -0.6471 to 0.5042 -0.7162 to 0.6066 -1.815 to 1.513 -0.5173 to 0.5507 -1.686 to 1.526 -1.181 to 0.9882 | 0.9652 0.9889 0.9804 0.9994 0.9966 0.9815 | 0.2686 |
| MCHC [g/dl] | 33.66  (n= 7) | 33.48  (n= 6) | 33.57  (n= 6) | 32.98  (n= 5) | t0 vs. t7 t0 vs. t20  t0 vs. t40  t7 vs. t20 t7 vs. t40 t20 vs. t40 | 0.1738 0.09048 0.6771 -0.08333 0.5033 0.5867 | -0.4280 to 0.7756 -1.174 to 1.355 -1.295 to 2.649 -1.176 to 1.009 -1.246 to 2.253 -0.4172 to 1.591 | 0.7229 0.9928 0.5606 0.9913 0.6726 0.2233 | 0.5791 |
| MCV [fl] | 87.56  (n= 7) | 88.03  (n= 6) | 87.85  (n= 6) | 89.6  (n= 5) | t0 vs. t7 t0 vs. t20  t0 vs. t40  t7 vs. t20 t7 vs. t40 t20 vs. t40 | -0.4762 -0.2929 -2.043 0.1833 -1.567 -1.750 | -2.375 to 1.422 -2.738 to 2.152 -7.053 to 2.967 -2.468 to 2.835 -5.610 to 2.477 -5.488 to 1.988 | 0.7938 0.9684 0.4441 0.9934 0.4789 0.3523 | 0.6342 |
| Erythrocyte distribution [%] | 13.03  (n= 7) | 13.12  (n= 6) | 13.23  (n= 6) | 13.54  (n= 5) | t0 vs. t7 t0 vs. t20  t0 vs. t40  t7 vs. t20 t7 vs. t40 t20 vs. t40 | -0.08810 -0.2048 -0.6114 -0.1167 -0.5233 -0.4067 | -0.5083 to 0.3321 -1.022 to 0.6123 -1.873 to 0.6505 -0.7298 to 0.4965 -1.711 to 0.6644 -0.9459 to 0.1326 | 0.8634 0.7942 0.3306 0.8922 0.3920 0.1168 | 0.06033 |
| Microcytic erythrocytes [%] | 1.733  (n= 7) | 1.6  (n= 6) | 1.8  (n= 6) | 1.54  (n= 5) | t0 vs. t7 t0 vs. t20  t0 vs. t40  t7 vs. t20 t7 vs. t40 t20 vs. t40 | 0.1333 -0.06667 0.1933 -0.2000 0.06000 0.2600 | -0.7483 to 1.015 -0.9204 to 0.7871 -1.298 to 1.684 -0.8669 to 0.4669 -0.2951 to 0.4151 -0.3697 to 0.8897 | 0.9220 0.9873 0.9175 0.7016 0.8968 0.4356 | 0.5228 |
| Granulocyte count (absolute) [10^3/µl] | 3.973  (n= 7) | 2.973  (n= 6) | 2.247  (n= 6) | 2.58  (n= 5) | t0 vs. t7 t0 vs. t20  t0 vs. t40  t7 vs. t20 t7 vs. t40 t20 vs. t40 | 0.9995 1.726 1.393 0.7267 0.3933 -0.3333 | -0.1632 to 2.162 0.2519 to 3.200 0.1270 to 2.659 -0.2770 to 1.730 -1.405 to 2.192 -1.632 to 0.9652 | 0.0847 **0.0275 0.0366** 0.1446 0.8114 0.7362 | 0.6723 |
| Granulocyte count (relative)  [%] | 59.19  (n= 7) | 47.88  (n= 6) | 42.9  (n= 6) | 44.76  (n= 5) | t0 vs. t7 t0 vs. t20  t0 vs. t40  t7 vs. t20 t7 vs. t40 t20 vs. t40 | 11.30 16.39 14.43 5.083 3.123 -1.960 | -4.235 to 26.84 -1.401 to 34.17 0.07560 to 28.78 -1.329 to 11.50 -14.98 to 21.23 -16.65 to 12.73 | 0.1427 0.0670 0.0492 0.1100 0.8915 0.9438 | 0.5243 |
| Lymphocyte count (absolute) [10^3/µl] | 1.931  (n= 7) | 2.357  (n= 6) | 2.15  (n= 6) | 2.272  (n= 5) | t0 vs. t7 t0 vs. t20  t0 vs. t40  t7 vs. t20 t7 vs. t40 t20 vs. t40 | -0.4252 -0.2186 -0.3406 0.2067 0.08467 -0.1220 | -1.104 to 0.2531 -0.6796 to 0.2425 -1.394 to 0.7125 -0.3160 to 0.7293 -0.5868 to 0.7562 -0.7574 to 0.5134 | 0.2139 0.3911 0.6000 0.5205 0.9516 0.8596 | 0.5647 |
| Lymphocyte count (relative) [%] | 28.47  (n= 7) | 39.55  (n= 6) | 42.38  (n= 6) | 39.62  (n= 5) | t0 vs. t7 t0 vs. t20  t0 vs. t40  t7 vs. t20 t7 vs. t40 t20 vs. t40 | -11.08 -13.91 -11.15 -2.833 -0.07000 2.763 | -26.86 to 4.700 -28.97 to 1.144 -22.80 to 0.5016 -12.34 to 6.673 -11.95 to 11.81 -5.186 to 10.71 | 0.1579 0.0663 0.0575 0.7052 >0.9999 0.5524 | 0.4493 |
| Eosinophil count (absolute) [10^3/µl] | 0.2257  (n= 7) | 0.1883  (n= 6) | 0.1117  (n= 6) | 0.3400  (n= 5) | t0 vs. t7 t0 vs. t20  t0 vs. t40  t7 vs. t20 t7 vs. t40 t20 vs. t40 | 0.03738 0.1140 -0.1143 0.07667 -0.1517 -0.2283 | -0.1604 to 0.2352 -0.01986 to 0.2480 -0.8349 to 0.6063 -0.1386 to 0.2920 -0.7785 to 0.4752 -0.9470 to 0.4904 | 0.8940 0.0874 0.9119 0.5932 0.7660 0.6115 | 0.4051 |
| Eosinophil count (relative) [%] | 3.371  (n= 7) | 3.067  (n= 6) | 2.25  (n= 6) | 5.180  (n= 5) | t0 vs. t7 t0 vs. t20  t0 vs. t40  t7 vs. t20 t7 vs. t40 t20 vs. t40 | 0.3048 1.121 -1.809 0.8167 -2.113 -2.930 | -2.818 to 3.427 -0.9135 to 3.156 -11.41 to 7.789 -3.122 to 4.756 -10.71 to 6.487 -12.37 to 6.505 | 0.9822 0.2898 0.8657 0.8670 0.7584 0.6259 | 0.4618 |
| Monocyte count (absolute) [10^3/µl] | 0.5443  (n= 7) | 0.5317  (n= 6) | 0.58  (n= 6) | 0.562  (n= 5) | t0 vs. t7 t0 vs. t20  t0 vs. t40  t7 vs. t20 t7 vs. t40 t20 vs. t40 | 0.01262 -0.03571 -0.01771 -0.04833 -0.03033 0.01800 | -0.2104 to 0.2356 -0.2625 to 0.1911 -0.2851 to 0.2497 -0.2439 to 0.1472 -0.2644 to 0.2037 -0.2735 to 0.3095 | 0.9964 0.9336 0.9921 0.8004 0.9479 0.9936 | 0.8804 |
| Monocyte count (relative) [%] | 8.071  (n= 7) | 8.5  (n= 6) | 11.37  (n= 6) | 9.440  (n= 5) | t0 vs. t7 t0 vs. t20  t0 vs. t40  t7 vs. t20 t7 vs. t40 t20 vs. t40 | -0.4286 -3.295 -1.369 -2.867 -0.9400 1.927 | -3.913 to 3.056 -7.848 to 1.257 -6.218 to 3.481 -6.960 to 1.226 -6.005 to 4.125 -6.477 to 10.33 | 0.9660 0.1447 0.6839 0.1591 0.8705 0.7910 | 0.6308 |
| Erythrocyte distribution width [fl] | 41.47  (n= 7) | 42.38  (n= 6) | 42.35  (n= 6) | 44.7  (n= 5) | t0 vs. t7 t0 vs. t20  t0 vs. t40  t7 vs. t20 t7 vs. t40 t20 vs. t40 | -0.9119 -0.8786 -3.229 0.03333 -2.317 -2.350 | -3.216 to 1.392 -5.167 to 3.410 -9.392 to 2.935 -2.780 to 2.846 -7.552 to 2.918 -5.824 to 1.124 | 0.5198 0.8707 0.2833 >0.9999 0.3891 0.1563 | 0.5274 |
| Platelet-large cell ratio [%] | 35.40  (n= 7) | 32.37  (n= 6) | 30.10  (n= 6) | 30.94  (n= 5) | t0 vs. t7 t0 vs. t20  t0 vs. t40  t7 vs. t20 t7 vs. t40 t20 vs. t40 | 3.033 5.300 4.460 2.267 1.427 -0.8400 | -0.9907 to 7.057 -1.291 to 11.89 -2.739 to 11.66 -2.476 to 7.009 -7.468 to 10.32 -8.982 to 7.302 | 0.1284 0.1052 0.1947 0.3854 0.9094 0.9721 | 0.09343 |
| Basophil count (absolute) [10^3/µl] | 0.06143  (n= 7) | 0.06000  (n= 6) | 0.06333  (n= 6) | 0.05600  (n= 5) | t0 vs. t7 t0 vs. t20  t0 vs. t40  t7 vs. t20 t7 vs. t40 t20 vs. t40 | 0.001429 -0.001905 0.005429 -0.003333 0.004000 0.007333 | -0.01863 to 0.02149 -0.08304 to 0.07923 -0.02997 to 0.04083 -0.08784 to 0.08117 -0.02861 to 0.03661 -0.09665 to 0.1113 | 0.9929 0.9997 0.9192 0.9988 0.9551 0.9905 | 0.5041 |
| Basophil count (relative) [%] | 0.9  (n= 7) | 1.0  (n= 6) | 1.2  (n= 6) | 1.0  (n= 5) | t0 vs. t7 t0 vs. t20  t0 vs. t40  t7 vs. t20 t7 vs. t40 t20 vs. t40 | -0.1000 -0.3000 -0.1000 -0.2000 0.000 0.2000 | -0.4045 to 0.2045 -1.545 to 0.9453 -0.5297 to 0.3297 -1.632 to 1.232 -0.5648 to 0.5648 -1.536 to 1.936 | 0.6463 0.8114 0.7843 0.9519 >0.9999 0.9621 | 0.4781 |
| Immature granulocyte count [%] | 0.2143  (n= 7) | 0.2667  (n= 6) | 0.5500  (n= 6) | 0.4200  (n= 5) | t0 vs. t7 t0 vs. t20  t0 vs. t40  t7 vs. t20 t7 vs. t40 t20 vs. t40 | -0.05238 -0.3357 -0.2057 -0.2833 -0.1533 0.1300 | -0.1332 to 0.02847 -1.331 to 0.6596 -0.6032 to 0.1918 -1.342 to 0.7751 -0.5739 to 0.2672 -1.088 to 1.348 | 0.1965 0.6292 0.2904 0.7629 0.5203 0.9693 | 0.4003 |
| Normoblast count [%] | 0.0  (n= 7) | 0.0  (n= 6) | 0.0  (n= 6) | 0.0  (n= 5) |  |  |  |  |  |
| Mean platelet volume [fl] | 11.33  (n= 7) | 10.93  (n= 6) | 10.6  (n= 6) | 10.78  (n= 5) | t0 vs. t7 t0 vs. t20  t0 vs. t40  t7 vs. t20 t7 vs. t40 t20 vs. t40 | 0.3952 0.7286 0.5486 0.3333 0.1533 -0.1800 | -0.2073 to 0.9978 -0.1883 to 1.645 -0.4319 to 1.529 -0.3902 to 1.057 -1.029 to 1.336 -0.9778 to 0.6178 | 0.1903 0.1092 0.2462 0.4112 0.9479 0.7981 | 0.1241 |
| Quick [%] | 99.71  (n= 7) | 98.83  (n= 6) | 99.33  (n= 6) | 98.8  (n= 5) | t0 vs. t7 t0 vs. t20  t0 vs. t40  t7 vs. t20 t7 vs. t40 t20 vs. t40 | 0.8810 0.3810 0.9143 -0.5000 0.03333 0.5333 | -3.656 to 5.418 -0.8067 to 1.569 -2.108 to 3.937 -5.868 to 4.868 -5.167 to 5.234 -1.047 to 2.114 | 0.8867 0.6612 0.6424 0.9845 >0.9999 0.5722 | 0.3911 |
| INR | 0.9343  (n= 7) | 0.9683  (n= 6) | 0.9567  (n= 6) | 0.9420  (n= 5) | t0 vs. t7 t0 vs. t20  t0 vs. t40  t7 vs. t20 t7 vs. t40 t20 vs. t40 | -0.03405 -0.02238 -0.007714 0.01167 0.02633 0.01467 | -0.1308 to 0.06266 -0.07201 to 0.02724 -0.06226 to 0.04683 -0.06053 to 0.08386 -0.07201 to 0.1247 -0.03245 to 0.06178 | 0.6009 0.4264 0.9344 0.9290 0.7136 0.6244 | 0.4499 |
| aPTT [s] | 29.0  (n= 7) | 29.68  (n= 6) | 29.1  (n= 6) | 29.78  (n= 5) | t0 vs. t7 t0 vs. t20  t0 vs. t40  t7 vs. t20 t7 vs. t40 t20 vs. t40 | -0.6833 -0.1000 -0.7800 0.5833 -0.09667 -0.6800 | -3.621 to 2.255 -2.587 to 2.387 -2.339 to 0.7790 -4.048 to 5.215 -4.576 to 4.382 -2.797 to 1.437 | 0.8258 0.9987 0.3107 0.9637 0.9997 0.6043 | 0.3567 |
| Thrombin clotting time [s] | 16.69  (n= 7) | 16.97  (n= 6) | 17.78  (n= 6) | 17.42  (n= 5) | t0 vs. t7 t0 vs. t20  t0 vs. t40  t7 vs. t20 t7 vs. t40 t20 vs. t40 | -0.2810 -1.098 -0.7343 -0.8167 -0.4533 0.3633 | -1.192 to 0.6302 -2.565 to 0.3701 -2.321 to 0.8519 -2.172 to 0.5388 -1.732 to 0.8256 -2.126 to 2.853 | 0.6852 0.1315 0.3596 0.2360 0.5394 0.9288 | 0.4246 |
| Fibrinogen [mg/dl] | 386.6  (n= 7) | 353.8  (n= 6) | 336.0  (n= 5) | 335.6  (n= 5) | t0 vs. t7 t0 vs. t20  t0 vs. t40  t7 vs. t20 t7 vs. t40 t20 vs. t40 | 32.74 50.57 50.97 17.83 18.23 0.4000 | -72.09 to 137.6 -160.0 to 261.1 -79.73 to 181.7 -218.5 to 254.2 -130.2 to 166.6 -109.7 to 110.5 | 0.6776 0.7695 0.4745 0.9885 0.9549 >0.9999 | 0.5174 |
| D-dimer [ng/ml] | (n= 7) | (n= 6) | (n= 6) | (n=5) | t0 vs. t7 t0 vs. t20  t0 vs. t40  t7 vs. t20 t7 vs. t40 t20 vs. t40 | -33.48 -268.8 -169.1 -235.3 -135.7 99.67 | -678.5 to 611.5 -1807 to 1269 -1956 to 1618 -1219 to 748.7 -1394 to 1123 -1038 to 1237 | 0.9972 0.9130 0.9780 0.8145 0.9684 0.9823 | 0.4392 |
| Protein C activity [%] | 132.0  (n= 7) | 125.7  (n= 6) | 126.5  (n= 6) | 134.6  (n=5) | t0 vs. t7 t0 vs. t20  t0 vs. t40  t7 vs. t20 t7 vs. t40 t20 vs. t40 | 6.333 5.500 -2.600 -0.8333 -8.933 -8.100 | -30.26 to 42.93 -15.25 to 26.25 -25.88 to 20.68 -27.23 to 25.56 -47.06 to 29.20 -32.39 to 16.19 | 0.9152 0.7677 0.9652 0.9994 0.7812 0.5800 | 0.5983 |
| Protein S (free antigen) [%] | 117.6  (n= 7) | 109.7  (n= 6) | 107.2  (n= 6) | 120.0  (n=5) | t0 vs. t7 t0 vs. t20  t0 vs. t40  t7 vs. t20 t7 vs. t40 t20 vs. t40 | 7.905 10.40 -2.429 2.500 -10.33 -12.83 | -32.82 to 48.63 -17.55 to 38.36 -43.81 to 38.95 -17.70 to 22.70 -32.70 to 12.04 -41.82 to 16.15 | 0.8868 0.5630 0.9945 0.9654 0.3610 0.3888 | 0.4607 |
| Lupus anticoagulant screening [s] | 36.57  (n= 7) | 35.45  (n= 6) | 37.72  (n= 6) | 37.84  (n=5) | t0 vs. t7 t0 vs. t20  t0 vs. t40  t7 vs. t20 t7 vs. t40 t20 vs. t40 | 1.121 -1.145 -1.269 -2.267 -2.390 -0.1233 | -3.201 to 5.444 -5.831 to 3.541 -4.344 to 1.807 -9.662 to 5.128 -9.012 to 4.232 -2.995 to 2.749 | 0.7781 0.8053 0.4364 0.6888 0.5272 0.9978 | 0.5406 |
| Lupus anticoagulant PTT [s] | 35.63  (n= 7) | 34.98  (n= 6) | 35.20  (n= 6) | 35.36  (n=5) | t0 vs. t7 t0 vs. t20  t0 vs. t40  t7 vs. t20 t7 vs. t40 t20 vs. t40 | 0.6452 0.4286 0.2686 -0.2167 -0.3767 -0.1600 | -4.059 to 5.349 -3.561 to 4.418 -4.771 to 5.308 -6.560 to 6.127 -6.354 to 5.600 -2.418 to 2.098 | 0.9541 0.9767 0.9958 0.9992 0.9932 0.9904 | 0.5535 |
| Lupus anticoagulant ICA (Index of circulating anticoagulant) [s] | 59.19  (n= 7) | 53.82  (n= 6) | 56.12  (n= 6) | 56.98  (n=5) | t0 vs. t7 t0 vs. t20  t0 vs. t40  t7 vs. t20 t7 vs. t40 t20 vs. t40 | 5.369 3.069 2.206 -2.300 -3.163 -0.8633 | -14.20 to 24.94 -8.503 to 14.64 -18.46 to 22.87 -21.46 to 16.86 -12.78 to 6.448 -18.60 to 16.87 | 0.7504 0.7675 0.9693 0.9682 0.5886 0.9968 | 0.6349 |
| Lupus anticoagulant ICA (Index of circulating anticoagulant) 1+1 [s] | 53.46  (n= 7) | 52.28  (n= 6) | 53.42  (n= 6) | 54.42  (n=5) | t0 vs. t7 t0 vs. t20  t0 vs. t40  t7 vs. t20 t7 vs. t40 t20 vs. t40 | 1.174 0.04048 -1.063 -1.133 -2.237 -1.103 | -13.63 to 15.98 -7.679 to 7.760 -12.97 to 10.85 -15.52 to 13.25 -9.077 to 4.604 -9.430 to 7.223 | 0.9902 >0.9999 0.9814 0.9904 0.5929 0.9447 | 0.5777 |
| Lupus anticoagulant ICA NP [s] | 50.83  (n= 7) | 53.85  (n= 6) | 50.9  (n= 6) | 54.12  (n=5) | t0 vs. t7 t0 vs. t20  t0 vs. t40  t7 vs. t20 t7 vs. t40 t20 vs. t40 | -3.021 -0.07143 -3.291 2.950 -0.2700 -3.220 | -8.935 to 2.892 -2.292 to 2.149 -5.947 to -0.6360 -3.101 to 9.001 -6.132 to 5.592 -5.676 to -0.7639 | 0.3395 0.9993 **0.0244** 0.3715 0.9973 **0.0201** | 0.4491 |
| Cardiolipin-IgG-antibody [GPL U/ml] | 3.957  (n= 7) | 4.367  (n= 6) | 4.0  (n= 6) | 3.480  (n=5) | t0 vs. t7 t0 vs. t20  t0 vs. t40  t7 vs. t20 t7 vs. t40 t20 vs. t40 | -0.4095 -0.04286 0.4771 0.3667 0.8867 0.5200 | -5.385 to 4.566 -2.612 to 2.527 -2.379 to 3.334 -2.397 to 3.131 -3.775 to 5.548 -2.051 to 3.091 | 0.9891 >0.9999 0.8997 0.9581 0.8627 0.8416 | 0.2935 |
| Cardiolipin-IgM-antibody [MPL U/ml] | 1.486  (n= 7) | 1.917  (n= 6) | 1.6  (n= 6) | 1.68  (n=5) | t0 vs. t7 t0 vs. t20  t0 vs. t40  t7 vs. t20 t7 vs. t40 t20 vs. t40 | -0.4310 -0.1143 -0.1943 0.3167 0.2367 -0.08000 | -1.843 to 0.9811 -1.590 to 1.362 -2.746 to 2.357 -0.1242 to 0.7576 -1.072 to 1.545 -1.182 to 1.022 | 0.6913 0.9909 0.9882 0.1480 0.8782 0.9897 | 0.0006688 |
| β2-glycoprotein-IgG antibody [U/ml] | 2.514  (n= 7) | 2.417  (n= 6) | 2.550  (n= 6) | 1.640  (n=5) | t0 vs. t7 t0 vs. t20  t0 vs. t40  t7 vs. t20 t7 vs. t40 t20 vs. t40 | 0.09762 -0.03571 0.8743 -0.1333 0.7767 0.9100 | -3.413 to 3.609 -1.611 to 1.539 -1.123 to 2.872 -2.511 to 2.244 -3.541 to 5.094 -0.8813 to 2.701 | 0.9996 0.9998 0.3963 0.9965 0.8798 0.3015 | 0.5939 |
| β2-glycoprotein-IgM antibody [U/ml] | 0.6143  (n= 7) | 2.683  (n= 6) | 0.8  (n= 6) | 1.660  (n=5) | t0 vs. t7 t0 vs. t20  t0 vs. t40  t7 vs. t20 t7 vs. t40 t20 vs. t40 | -2.069 -0.1857 -1.046 1.883 1.023 -0.8600 | -9.861 to 5.723 -0.8208 to 0.4493 -2.819 to 0.7275 -5.969 to 9.735 -7.886 to 9.933 -2.735 to 1.015 | 0.7669 0.7159 0.2186 0.8133 0.9624 0.3657 | 0.3368 |
| Platelet aggregation ADP [%] | 71.71  (n= 7) | 73.80  (n= 5) | 70.17  (n= 6) | 64.6  (n=5) | t0 vs. t7 t0 vs. t20  t0 vs. t40  t7 vs. t20 t7 vs. t40 t20 vs. t40 | -2.086 1.548 7.114 3.633 9.200 5.567 | -21.95 to 17.78 -10.90 to 13.99 -29.21 to 43.44 -10.63 to 17.90 -19.70 to 38.10 -29.41 to 40.55 | 0.9706 0.9649 0.8530 0.7404 0.5167 0.9112 | 0.4379 |
| Platelet Aggrgation collagen [%] | 69.14  (n= 7) | 69.0  (n= 5) | 67.17  (n= 6) | 66.8  (n=5) | t0 vs. t7 t0 vs. t20  t0 vs. t40  t7 vs. t20 t7 vs. t40 t20 vs. t40 | 0.1429 1.976 2.343 1.833 2.200 0.3667 | -16.10 to 16.39 -9.471 to 13.42 -20.15 to 24.83 -5.603 to 9.270 -28.27 to 32.67 -26.64 to 27.37 | >0.9999 0.9157 0.9713 0.7568 0.9829 >0.9999 | 0.5231 |
| Platelet aggregation Ristocetin 1,2 [%] | 70.86  (n= 7) | 69.8  (n= 5) | 72.0  (n= 6) | 66.8  (n=5) | t0 vs. t7 t0 vs. t20  t0 vs. t40  t7 vs. t20 t7 vs. t40 t20 vs. t40 | 1.057 -1.143 4.057 -2.200 3.000 5.200 | -10.14 to 12.25 -9.958 to 7.673 -24.33 to 32.44 -11.11 to 6.713 -26.67 to 32.67 -22.13 to 32.53 | 0.9782 0.9607 0.9326 0.7562 0.9567 0.8626 | 0.4214 |
| Platelet aggreagtion Ristocetin 0,6 [%] | 2.0  (n= 7) | 7.6  (n= 5) | 2.833  (n= 6) | 4.6  (n=5) | t0 vs. t7 t0 vs. t20  t0 vs. t40  t7 vs. t20 t7 vs. t40 t20 vs. t40 | -5.600 -0.8333 -2.600 4.767 3.000 -1.767 | -21.08 to 9.875 -5.552 to 3.885 -11.23 to 6.027 -14.48 to 24.02 -3.710 to 9.710 -12.63 to 9.095 | 0.5254 0.9107 0.6446 0.7546 0.3108 0.9062 | 0.5362 |
| Platelet aggregometry (PFA)  Collagen/epinephrin [s] | 150.9  (n= 7) | 175.8  (n= 6) | 144.0  (n= 6) | 182.6  (n=5) | t0 vs. t7 t0 vs. t20  t0 vs. t40  t7 vs. t20 t7 vs. t40 t20 vs. t40 | -24.98 6.857 -31.74 31.83 -6.767 -38.60 | -92.67 to 42.71 -94.58 to 108.3 -80.14 to 16.65 -69.95 to 133.6 -112.2 to 98.70 -154.0 to 76.78 | 0.5689 0.9939 0.1691 0.6767 0.9928 0.5779 | 0.4875 |
| Platelet aggregometry (PFA)  Collagen/ADP [s] | 117.9  (n= 7) | 101.0  (n= 6) | 87.5  (n= 6) | 102.4  (n=5) | t0 vs. t7 t0 vs. t20  t0 vs. t40  t7 vs. t20 t7 vs. t40 t20 vs. t40 | 16.86 30.36 15.46 13.50 -1.400 -14.90 | -22.92 to 56.64 -22.38 to 83.09 -47.06 to 77.98 -9.578 to 36.58 -24.83 to 22.03 -45.51 to 15.71 | 0.4710 0.2629 0.7554 0.2532 0.9942 0.3277 | 0.5368 |
| EXTEM Clotting Time (CT) [s] | 66.0  (n= 7) | 68.33  (n= 6) | 69.5  (n= 6) | 65.4  (n=5) | t0 vs. t7 t0 vs. t20  t0 vs. t40  t7 vs. t20 t7 vs. t40 t20 vs. t40 | -2.333 -3.500 0.6000 -1.167 2.933 4.100 | -20.32 to 15.65 -11.17 to 4.167 -8.807 to 10.01 -23.76 to 21.43 -26.34 to 32.20 -9.987 to 18.19 | 0.9606 0.4178 0.9929 0.9972 0.9742 0.6657 | 0.4463 |
| EXTEM clot formation time (CFT) [s] | 72.57  (n= 7) | 64.67  (n= 6) | 77.33  (n= 6) | 74.2  (n=5) | t0 vs. t7 t0 vs. t20  t0 vs. t40  t7 vs. t20 t7 vs. t40 t20 vs. t40 | 7.905 -4.762 -1.629 -12.67 -9.533 3.133 | -8.445 to 24.25 -30.94 to 21.42 -38.72 to 35.46 -43.94 to 18.61 -56.91 to 37.85 -51.79 to 58.06 | 0.3773 0.9037 0.9976 0.5034 0.8435 0.9949 | 0.6176 |
| EXTEM alpha [°] | 75.57  (n= 7) | 77.0  (n= 6) | 74.5  (n= 6) | 75.2  (n=5) | t0 vs. t7 t0 vs. t20  t0 vs. t40  t7 vs. t20 t7 vs. t40 t20 vs. t40 | -1.429 1.071 0.3714 2.500 1.800 -0.7000 | -4.781 to 1.924 -4.405 to 6.548 -7.058 to 7.801 -4.354 to 9.354 -7.207 to 10.81 -11.53 to 10.13 | 0.4671 0.8845 0.9965 0.5768 0.8459 0.9927 | 0.6463 |
| EXTEM A10 [mm] | 60.29  (n= 7) | 63.17  (n= 6) | 57.33  (n= 6) | 59.2  (n=5) | t0 vs. t7 t0 vs. t20  t0 vs. t40  t7 vs. t20 t7 vs. t40 t20 vs. t40 | -2.881 2.952 1.086 5.833 3.967 -1.867 | -8.000 to 2.238 -3.721 to 9.626 -8.791 to 10.96 -2.440 to 14.11 -11.50 to 19.43 -16.77 to 13.04 | 0.2767 0.4402 0.9667 0.1561 0.7367 0.9525 | 0.6324 |
| EXTEM A20 [mm] | 66.29  (n= 7) | 69.0  (n= 6) | 63.83  (n= 6) | 65.0  (n=5) | t0 vs. t7 t0 vs. t20  t0 vs. t40  t7 vs. t20 t7 vs. t40 t20 vs. t40 | -2.714 2.452 1.286 5.167 4.000 -1.167 | -7.188 to 1.759 -2.491 to 7.396 -6.424 to 8.996 -1.665 to 12.00 -8.356 to 16.36 -11.70 to 9.369 | 0.2320 0.3595 0.9001 0.1271 0.5994 0.9660 | 0.6611 |
| EXTEM maximum clot firmness (MCF) [mm] | 67.57  (n= 7) | 70.33  (n= 6) | 66.33  (n= 6) | 67.0  (n=5) | t0 vs. t7 t0 vs. t20  t0 vs. t40  t7 vs. t20 t7 vs. t40 t20 vs. t40 | -2.762 1.238 0.5714 4.000 3.333 -0.6667 | -6.374 to 0.8502 -3.115 to 5.591 -7.116 to 8.259 -2.391 to 10.39 -7.710 to 14.38 -10.26 to 8.929 | 0.1230 0.7313 0.9890 0.2148 0.6436 0.9909 | 0.6320 |
| EXTEM maximum lysis (ML) [°] | 6.571  (n= 7) | 4.5  (n= 6) | 3.0  (n= 6) | 3.2  (n=5) | t0 vs. t7 t0 vs. t20  t0 vs. t40  t7 vs. t20 t7 vs. t40 t20 vs. t40 | 2.071 3.571 3.371 1.500 1.300 -0.2000 | -2.051 to 6.193 0.08659 to 7.056 -2.391 to 9.133 -0.07993 to 3.080 -1.725 to 4.325 -2.262 to 1.862 | 0.3507 **0.0457** 0.2226 0.0603 0.4087 0.9765 | 0.4940 |
| INTEM Clotting Time (CT) [s] | 166.7  (n= 7) | 176.8  (n= 6) | 163.5  (n= 6) | 161.2  (n=5) | t0 vs. t7 t0 vs. t20  t0 vs. t40  t7 vs. t20 t7 vs. t40 t20 vs. t40 | -10.12 3.214 5.514 13.33 15.63 2.300 | -37.61 to 17.37 -22.24 to 28.66 -24.73 to 35.76 -28.71 to 55.38 -27.38 to 58.64 -16.69 to 21.29 | 0.5705 0.9634 0.8758 0.6682 0.5224 0.9566 | 0.5585 |
| INTEM alpha [°] | 78.14  (n= 7) | 79.5  (n= 6) | 78.33  (n= 6) | 78.2  (n=5) | t0 vs. t7 t0 vs. t20  t0 vs. t40  t7 vs. t20 t7 vs. t40 t20 vs. t40 | -1.357 -0.1905 -0.05714 1.167 1.300 0.1333 | -5.153 to 2.439 -4.211 to 3.830 -4.989 to 4.875 -5.731 to 8.064 -5.317 to 7.917 -8.254 to 8.521 | 0.5905 0.9978 >0.9999 0.9200 0.8519 0.9999 | 0.7259 |
| INTEM A10 [mm] | 60.29  (n= 7) | 62.33  (n= 6) | 58.17  (n= 6) | 58.8  (n=5) | t0 vs. t7 t0 vs. t20  t0 vs. t40  t7 vs. t20 t7 vs. t40 t20 vs. t40 | -2.048 2.119 1.486 4.167 3.533 -0.6333 | -7.016 to 2.921 -3.290 to 7.528 -7.245 to 10.22 -2.531 to 10.86 -8.706 to 15.77 -12.26 to 10.99 | 0.4910 0.5270 0.8950 0.2181 0.6706 0.9955 | 0.7408 |
| INTEM A20 [mm] | 64.71  (n= 7) | 67.33  (n= 6) | 64.17  (n= 6) | 64.2  (n=5) | t0 vs. t7 t0 vs. t20  t0 vs. t40  t7 vs. t20 t7 vs. t40 t20 vs. t40 | -2.619 0.5476 0.5143 3.167 3.133 -0.03333 | -6.740 to 1.502 -4.003 to 5.098 -7.447 to 8.475 -2.173 to 8.507 -6.730 to 13.00 -9.490 to 9.423 | 0.2066 0.9680 0.9927 0.2452 0.6115 >0.9999 | 0.7192 |
| INTEM maximum clot firmness (MCF) [mm] | 65.0  (n= 7) | 68.0  (n= 6) | 65.0  (n= 6) | 64.8  (n=5) | t0 vs. t7 t0 vs. t20  t0 vs. t40  t7 vs. t20 t7 vs. t40 t20 vs. t40 | -3.000 0.000 0.2000 3.000 3.200 0.2000 | -6.895 to 0.8951 -3.672 to 3.672 -7.347 to 7.747 -2.041 to 8.041 -6.668 to 13.07 -8.705 to 9.105 | 0.1203 >0.9999 0.9995 0.2431 0.5983 0.9997 | 0.6205 |
| INTEM maximum lysis (ML) [°] | 8.286  (n= 7) | 6.667  (n= 6) | 5.167  (n= 6) | 5.8  (n=5) | t0 vs. t7 t0 vs. t20  t0 vs. t40  t7 vs. t20 t7 vs. t40 t20 vs. t40 | 1.619 3.119 2.486 1.500 0.8667 -0.6333 | -1.147 to 4.385 -0.4753 to 6.713 -3.195 to 8.166 -0.7846 to 3.785 -4.261 to 5.995 -4.304 to 3.037 | 0.2529 0.0821 0.3966 0.1898 0.8968 0.8914 | 0.6655 |
| FIBTEM Clotting Time (CT) [s] | 59.57  (n= 7) | 60.83  (n= 6) | 65.33  (n= 6) | 61.0  (n=5) | t0 vs. t7 t0 vs. t20  t0 vs. t40  t7 vs. t20 t7 vs. t40 t20 vs. t40 | -1.262 -5.762 -1.429 -4.500 -0.1667 4.333 | -9.975 to 7.451 -20.84 to 9.318 -14.81 to 11.96 -19.53 to 10.53 -13.73 to 13.39 -7.037 to 15.70 | 0.9469 0.5447 0.9693 0.7024 >0.9999 0.4902 | 0.7234 |
| FIBTEM alpha [°] | 75.0  (n= 7) | 76.83  (n= 6) | 76.6  (n= 5) | 75.6  (n=5) | t0 vs. t7 t0 vs. t20  t0 vs. t40  t7 vs. t20 t7 vs. t40 t20 vs. t40 | -1.833 -1.600 -0.6000 0.2333 1.233 1.000 | -5.068 to 1.402 -7.303 to 4.103 -11.38 to 10.18 -4.142 to 4.609 -10.14 to 12.61 -7.075 to 9.075 | 0.2724 0.6874 0.9953 0.9958 0.9679 0.9265 | 0.4092 |
| FIBTEM A10 [mm] | 19.86  (n= 7) | 23.5  (n= 6) | 20.83  (n= 6) | 20.6  (n=5) | t0 vs. t7 t0 vs. t20  t0 vs. t40  t7 vs. t20 t7 vs. t40 t20 vs. t40 | -3.643 -0.9762 -0.7429 2.667 2.900 0.2333 | -8.082 to 0.7966 -10.58 to 8.626 -9.832 to 8.347 -10.07 to 15.40 -10.17 to 15.97 -6.688 to 7.155 | 0.0986 0.9801 0.9855 0.8638 0.8052 0.9989 | 0.4547 |
| FIBTEM A20 [mm] | 20.71  (n= 7) | 24.67  (n= 6) | 21.5  (n= 6) | 20.60  (n=5) | t0 vs. t7 t0 vs. t20  t0 vs. t40  t7 vs. t20 t7 vs. t40 t20 vs. t40 | -3.952 -0.7857 0.1143 3.167 4.067 0.9000 | -8.567 to 0.6626 -10.23 to 8.660 -9.090 to 9.319 -9.197 to 15.53 -9.149 to 17.28 -5.412 to 7.212 | 0.0858 0.9888 >0.9999 0.7842 0.6317 0.9330 | 0.4429 |
| FIBTEM maximum clot firmness (MCF) [mm] | 20.71  (n= 7) | 24.67  (n= 6) | 21.67  (n= 6) | 20.6  (n=5) | t0 vs. t7 t0 vs. t20  t0 vs. t40  t7 vs. t20 t7 vs. t40 t20 vs. t40 | -3.952 -0.9524 0.1143 3.000 4.067 1.067 | -8.039 to 0.1345 -10.57 to 8.668 -9.138 to 9.366 -9.495 to 15.49 -9.149 to 17.28 -4.841 to 6.975 | 0.0564 0.9815 >0.9999 0.8128 0.6317 0.8787 | 0.3939 |
| FIBTEM maximum lysis (ML) [°] | 1.429  (n= 7) | 2.333  (n= 6) | 1.333  (n= 6) | 1.2  (n=5) | t0 vs. t7 t0 vs. t20  t0 vs. t40  t7 vs. t20 t7 vs. t40 t20 vs. t40 | -0.9048 0.09524 0.2286 1.000 1.133 0.1333 | -5.403 to 3.593 -3.340 to 3.531 -4.281 to 4.738 -2.160 to 4.160 -1.922 to 4.188 -1.510 to 1.777 | 0.8764 0.9996 0.9964 0.6695 0.5085 0.9858 | 0.4849 |
| APTEM Clotting Time (CT) [s] | 61.0  (n= 7) | 65.67  (n= 6) | 63.33  (n= 6) | 59.4  (n=5) | t0 vs. t7 t0 vs. t20  t0 vs. t40  t7 vs. t20 t7 vs. t40 t20 vs. t40 | -4.667 -2.333 1.600 2.333 6.267 3.933 | -18.78 to 9.442 -14.20 to 9.537 -12.33 to 15.53 -12.01 to 16.68 -14.30 to 26.83 -10.22 to 18.09 | 0.6418 0.8832 0.9625 0.9277 0.6377 0.6927 | 0.7993 |
| APTEM clot formation time (CFT) [s] | 70.0  (n= 7) | 64.0  (n= 6) | 73.5  (n= 6) | 77.6  (n=5) | t0 vs. t7 t0 vs. t20  t0 vs. t40  t7 vs. t20 t7 vs. t40 t20 vs. t40 | 6.000 -3.500 -7.600 -9.500 -13.60 -4.100 | -9.225 to 21.22 -19.13 to 12.13 -45.12 to 29.92 -30.95 to 11.95 -54.30 to 27.10 -36.68 to 28.48 | 0.5228 0.8403 0.8411 0.4393 0.5788 0.9519 | 0.6793 |
| APTEM alpha [°] | 76.14  (n= 7) | 77.17  (n= 6) | 75.5  (n= 6) | 74.6  (n=5) | t0 vs. t7 t0 vs. t20  t0 vs. t40  t7 vs. t20 t7 vs. t40 t20 vs. t40 | -1.024 0.6429 1.543 1.667 2.567 0.9000 | -4.223 to 2.176 -1.923 to 3.209 -4.369 to 7.455 -1.855 to 5.189 -3.447 to 8.580 -3.616 to 5.416 | 0.6626 0.7943 0.7276 0.3924 0.4132 0.8469 | 0.6354 |
| APTEM A10 [mm] | 60.43  (n= 7) | 62.0  (n= 6) | 57.83  (n= 6) | 57.4  (n=5) | t0 vs. t7 t0 vs. t20  t0 vs. t40  t7 vs. t20 t7 vs. t40 t20 vs. t40 | -1.571 2.595 3.029 4.167 4.600 0.4333 | -5.378 to 2.235 -2.272 to 7.463 -8.827 to 14.88 -2.394 to 10.73 -8.432 to 17.63 -10.26 to 11.13 | 0.4898 0.3111 0.7387 0.2069 0.5422 0.9981 | 0.6110 |
| APTEM A20 [mm] | 66.29  (n= 7) | 67.67  (n= 6) | 63.67  (n= 6) | 63.4  (n=5) | t0 vs. t7 t0 vs. t20  t0 vs. t40  t7 vs. t20 t7 vs. t40 t20 vs. t40 | -1.381 2.619 2.886 4.000 4.267 0.2667 | -5.175 to 2.413 -1.175 to 6.413 -6.945 to 12.72 -1.389 to 9.389 -7.159 to 15.69 -8.136 to 8.669 | 0.5783 0.1656 0.6606 0.1345 0.5041 0.9991 | 0.5857 |
| APTEM maximum clot firmness (MCF) [mm] | 67.0  (n= 7) | 69.0  (n= 6) | 65.5  (n= 6) | 65.2  (n=5) | t0 vs. t7 t0 vs. t20  t0 vs. t40  t7 vs. t20 t7 vs. t40 t20 vs. t40 | -2.000 1.500 1.800 3.500 3.800 0.3000 | -5.180 to 1.180 -2.026 to 5.026 -7.331 to 10.93 -2.116 to 9.116 -6.966 to 14.57 -6.868 to 7.468 | 0.2122 0.4682 0.8507 0.2171 0.5422 0.9980 | 0.5151 |
| APTEM maximum lysis (ML) [°] | 7.143  (n= 7) | 4.833  (n= 6) | 3.667  (n= 6) | 3.8  (n=5) | t0 vs. t7 t0 vs. t20  t0 vs. t40  t7 vs. t20 t7 vs. t40 t20 vs. t40 | 2.310 3.476 3.343 1.167 1.033 -0.1333 | -0.8201 to 5.439 0.4492 to 6.503 -2.460 to 9.146 -1.428 to 3.761 -5.873 to 7.940 -5.013 to 4.747 | 0.1368 0.0296 0.2307 0.4286 0.9241 0.9994 | 0.6693 |
|  |  |  |  |  |  |  |  |  |  |
| Blood group | A (n= 1) | CC D ee | Kell neg. (n= 1) | Irregular antibodies (n= 0) |  |  |  |  |  |

^*^Significant *p*-values are written in bold font
